# Potent IgE-Mediated Allergenic Reduction in Human Serum by Casein–Polyphenol Conjugates Makes Personalized Oral Immunotherapy Credible

**DOI:** 10.1021/acsfoodscitech.6c00400

**Published:** 2026-07-29

**Authors:** M. Victoria Gil, Nuria Fernández-Rivera, Gloria Gutiérrez-Díaz, Jorge Parrón-Ballesteros, Carlos Pastor-Vargas, Diana Betancor, Carlos Nieto, Pedro Cintas

**Affiliations:** † Department of Organic and Inorganic Chemistry, Faculty of Sciences, IACYS-Unidad de Química Verde y Desarrollo Sostenible, 16759 Universidad de Extremadura, 06006 Badajoz, Spain; ‡ Department of Biochemistry and Molecular Biology, Faculty of Chemistry, Universidad Complutense de Madrid, 28040 Madrid, Spain; § Department of Allergy and Immunology, IIS-Fundación Jiménez Díaz, UAM, 28040 Madrid, Spain; ∥ Department of Organic Chemistry, Faculty of Chemical Sciences, 16779 Universidad de Salamanca, Pl. Caídos s/n, 37008 Salamanca, Spain

**Keywords:** chemically modified proteins, cow’s milk allergy, IgE-mediated anaphylaxis, oral immunotherapy, personalized medicine, polyphenols

## Abstract

This study describes a potent hypoallergenic casein–polyphenol formula based on preclinical assays of serum samples from patients allergic to cow’s milk proteins. Despite some variability among patients, results showed a significant IgE-binding reduction. This proof of concept indicates the reliability of altering the native structure and potential epitopes of allergens by natural polyphenols, consistent with previous studies monitoring amide bonding by vibrational spectroscopy. Structural predictions, binding pocket scouting, and molecular docking were used to explore the nature of the binding between polyphenols and casein isoforms. *In silico* modeling identified the interaction of polyphenol–protein hybrids with IgE-binding epitopes in α-S_1_, α-S_2_, and β-caseins, albeit only one in κ-casein, which provides a molecular basis for the clinically observed hypoallergenicity. Overall, preclinical analysis holds promise for oral immunotherapy to end food sensitivity and reduce the risk of life-threatening anaphylaxis. Parallel bioassays, however, with β-lactoglobulin, another cow’s milk allergen, afforded promising results.

## Graphical abstract

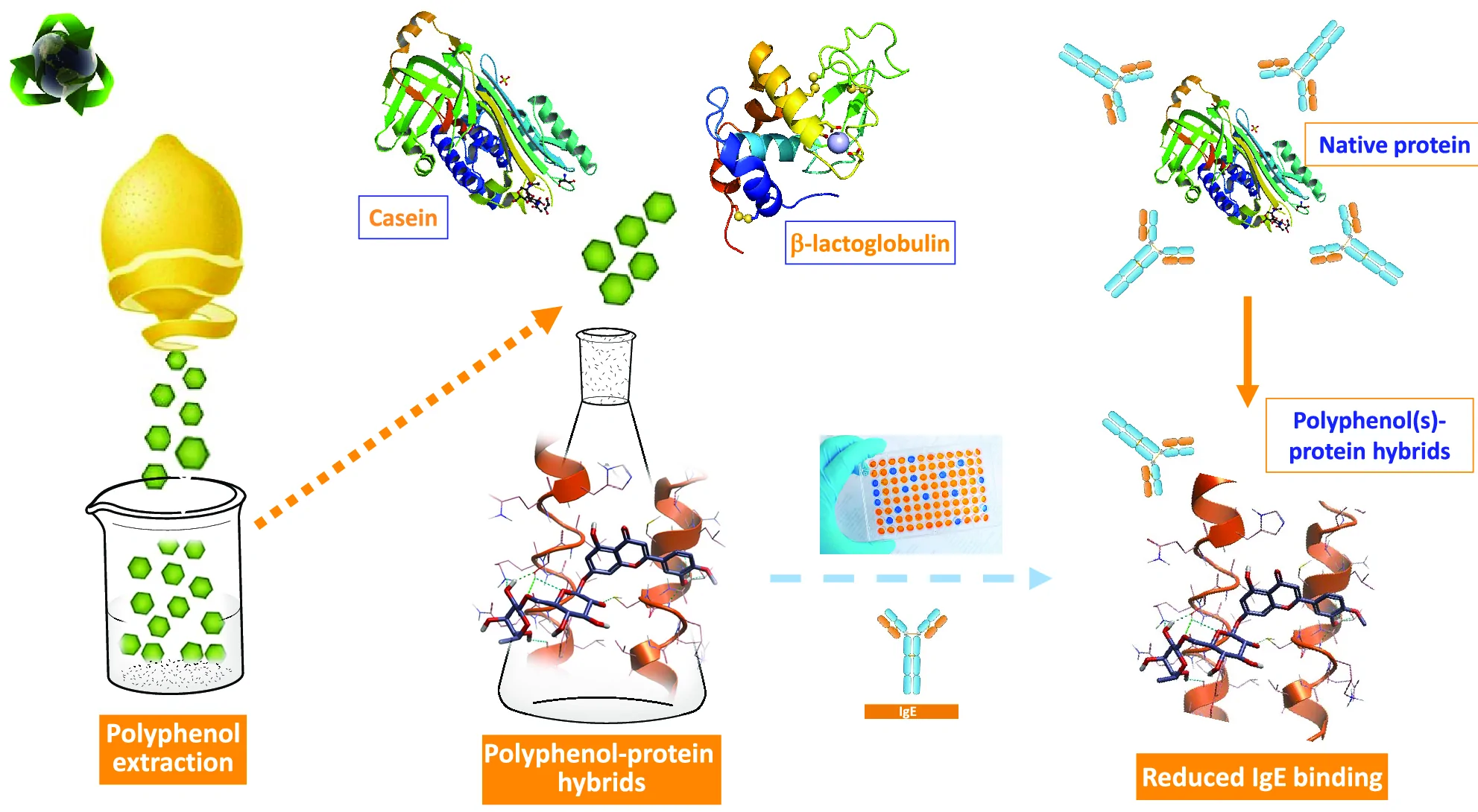

## 1 Introduction

Food allergy constitutes a global concern and an underestimated pandemic indeed.^1^ In the United States, roughly one in 10 adults (age 18 or older) and one in 13 children have at least one food allergy.^2^ IgE-mediated food sensitivity in children puts their lives at extraordinary risk when they consume something with a minuscule cross-contamination, often due to a manufacturing error, which unleashes multiple symptoms, ranging from local reactions to life-threatening anaphylaxis. Most food allergies are caused by nuts, peanuts, milk, eggs, fish, crustaceans, and some fruits. They contain complex proteins that remain intact during digestion and can trigger immunological responses. Food allergy is essentially an inflammatory process, whereby the immune system recognizes certain proteins as foes and triggers a sequential reaction involving the cross-linking of allergen-specific immunoglobulin E (IgE) on mast cells.^3,4^ How to keep people safe from allergies has been rethought since the late 1990s. International studies support the method of desensitization called oral immunotherapy (OIT), i.e., giving a small and escalating dose by mouth, although small doses of allergens may also be administered safely on the skin and sublingually.^5^ In 2020, Palforzia (trade name) was the first-ever FDA-approved therapy for peanut allergy, and recent early phase trials also involved the administration of monoclonal antibodies, which are FDA-approved drugs for treating other diseases like asthma. Such antibodies (e.g., dupilumab or linvoseltamab) appear to block the action of specific cytokines, which are signaling proteins launching the production of IgE.^6^ Moreover, these antibody-based therapeutics are supplemental to allergoid treatment and are not alternatives to allergoids themselves (see below).

Oral immunotherapy faces difficulties in ensuring dosage, as a negligible quantity for one individual may pose a severe risk to the other.^7^ Moreover, food-specific IgE levels do not necessarily correlate with the severity of symptoms or with potential responsiveness to OIT. Therefore, an individual assessment of the safety of OIT for every allergic patient is required, as well as the development of safer and more effective OIT candidates.

It is well-known that both physical and chemical treatments may significantly alter the native structure of proteins, leading to misfolded or inactive conformations,^8^ which represent a potentially useful strategy for OIT, making food allergens more tolerable to allergic patients. Conventional treatments such as thermal inactivation or enzymatic hydrolysis may reduce allergenicity, although protein functionality could be compromised as well.^9^ Chemical treatments often involve the formation of either covalent or noncovalent protein–ligand complexes through competition at active or allosteric positions. Overall, the effect is akin to the well-known protein denaturation that can be induced by small molecules.^10^ Flavonoids and polyphenols, in general, can exert strong antagonistic effects on protein hosts, including modulation of IgE-mediated activity,^11–14^ because of multiple hydrophobic and H-bonding interactions. Conventional allergoids, well portrayed by cross-linking agents such as glutaraldehyde,^15,16^ represent structural modifiers capable of altering specific Ig site binding. Moreover, analysis of the human interactome indicates that proximity effects of polyphenol protein targets and proteins linked to diseases predict novel molecular mechanisms for health.^17^

Early studies using polyphenol-fortified peanut proteins showed a reduction in IgE binding relative to control experiments, thereby producing hypoallergenic matrices that could be employed for OIT.^18^ Recently, we introduced, as a proof of concept, the generation of edible protein matrices containing polyphenols from orange peel targeting cow’s milk allergy. Unlike classical allergoids available in clinical practice, which reduce IgE binding by chemical modifications, we aimed to establish protein matrices formed by high-affinity noncovalent interactions with value-added molecules that impair IgE binding and promote tolerogenic responses mediated by polyphenols. Therefore, these compounds would promote allergen desensitization in patients resistant to conventional OIT, which is the case in adults allergic to cow’s milk.

Some of these compounds moderately decreased IgE binding in serum from patients sensitized to β-lactoglobulin, whereas the results for a major allergen like casein were poor.^19^ In this study, we employ lemon peels, with a distinctive range and contents of polyphenols, which indicate a remarkable disruption of IgE binding tested in human sera from patients with preexisting allergy to casein and β-lactoglobulin, especially the former. Protein–polyphenol complexes were generated through simple incubation of both components at different ratios and conditions. The use of pure polyphenols allowed us to determine their individual efficiency as well as the matrix effects associated with the natural extract. On the other hand, casein exhibits large structural variability, either in native or ligand-modified forms, as shown by results reported in PDB and other resources. This impedes the ability to foresee with confidence the putative casein substructures involved in allergic reactions for a given patient and hence model the corresponding epitope interactions. In any case, docking screening has been conducted on the conformational landscape accounting for the reduction of IgE binding. The approach lays the groundwork for the next generation of oral immunotherapeutics, favoring direct and personalized clinical translation.

## 2 Materials and Methods

### 2.1 Materials

Lemon fruits (*Citrus lemon* (L.) Burm. *ethnovar*. “fino”) were obtained from local markets. Fruits were successively washed and peeled (albedo and flavedo layers) and stored at −20 °C under vacuum prior to obtaining the polyphenolic extract. Casein and β-lactoglobulin (from bovine milk) and polyphenol standards were purchased from commercial suppliers (Sigma-Aldrich) and used as received.

### 2.2 Preparation of Phenolic Extracts

Polyphenol extraction was performed according to the method reported in the literature.^20^ Fruit peels, previously frozen at −20 °C, were placed inside a drying oven (P-Selecta 210) at 145 °C for 700 min and then triturated to produce a finely divided material (particle size: 0.3–0.5 mm) to facilitate the interaction with the aqueous phase. This solid material (25.00 ± 0.01 g) was suspended in Milli-Q-quality water (200 mL, 1:8 ratio) and the mixture was heated in a thermostated bath (P-Selecta, Unitronic OR) at 57 °C for 700 min under continuous stirring (97 rpm). After filtration, the resulting sample was centrifuged at 10,000 rpm for 10 min at 4 °C (Allegra 25R, Beckman Coulter) to separate any precipitate, then frozen at −80 °C, and lyophilized (Virtis Genesis 25 LL, SP Scientific). The resulting extracts were stored under refrigeration in the dark to minimize the sensitivity of polyphenols to degradation under ambient conditions.^21^

### 2.3 Quantification of Polyphenols

The total amount of polyphenolic compounds was determined by spectrophotometry according to a previously described method.^22^ The colorimetric assay was conducted by mixing the extract (0.25 mL), water (2 mL, Milli-Q quality), and the Folin–Ciocalteu reagent (phosphomolybdate–phosphotungstate mixture, 1 mL). After 3 min, a saturated aqueous solution of Na_2_CO_3_ (2 mL) was added, and the mixture was diluted to 25 mL (Milli-Q water). This redox transformation fits a proportional relationship between the polyphenol concentration and absorbance measured at 760 nm (UV-2450 Shimadzu Corporation spectrophotometer). The quantification of total polyphenols was expressed as milligrams of gallic acid per 100 g of sample (external standard method).

### 2.4 HPLC Analyses

Polyphenols in the lyophilizates of lemon peel were separated and identified by HPLC in agreement with literature protocols.^23^ To the lyophilized powder extract (0.5 g), a MeOH/H_2_O solution (1:1 v/v, 10 mL) containing NaF (2 mM) was added. Samples were subsequently sonicated (ultrasonic cleaning bath, 40 kHz nominal frequency) for 30 min at room temperature (cold water was added to maintain the bulk temperature), and sunlight exposure was avoided to minimize the degradation of polyphenols. The resulting extracts were centrifuged at 4000 rpm for 5 min at 4 °C. An aliquot (1 mL) of the supernatant was diluted to 5 mL using the aforementioned aqueous MeOH solution, and the sample was filtered through a 0.2 μm filter. HPLC analyses were carried out using an Agilent Technologies 1100 series apparatus equipped with a quaternary pump, vacuum degasser, automatic sampler, photodiode array, and fluorescence detectors. Samples were injected onto a C-18 Gemini-NX column (Phenomenex, 4.6 mm, 4.6 mm × 150 mm) thermostated at 40 °C. The mobile phase consisted of distilled water (A) and CH_3_CN (B), each containing 0.1% (v/v) HCOOH, using the following gradient: initially 3% B; 0–30 min, 35% B; 30–33 min, 50% B; 33–34 min, 100% B. The flow rate was set at 1.0 mL/min with the injection of 10 μL samples in all cases. Polyphenols were detected at 280, 320, 350, and 255 nm using the photodiode array, and by fluorescence detection with excitation at 275 nm and emission at 315 nm. The quantification of compounds was done by calibration curves using commercial external standards. Data processing was accomplished using HP software (ChemStation, Rev.B.04.01, Agilent Technologies).

### 2.5 Synthesis of Hybrid Protein–Polyphenol Matrices

Polyphenol–milk protein matrices were obtained according to the method described by Lila and associates for polyphenol-fortified peanut proteins.^18^ Protein solutions (5 g/L) were prepared in Milli-Q water. A solution of freeze-dried extract of citrus peel (30 g/L) was also prepared, from which 15 mL was taken and diluted in water to 100 mL, leading to a polyphenol stock solution (4.5 g/L) for obtaining the hybrid matrices. Thus, 20 mL of every protein solution (5 g/L) was combined with 10 mL solutions of polyphenol commercial standards. Assays were conducted at room temperature and with different protein:polyphenol ratios (2:1, 1:1, and 1:2 ratios). When pure polyphenols were used, a 2:1 protein:polyphenol ratio afforded the greatest interaction during matrix formation. The combined solutions were magnetically stirred for 30 min, avoiding sunlight exposure, followed by centrifugation at 12,000 rpm for 20 min. A control assay, involving proteins in only an aqueous solution, was obtained. The supernatant was decanted, and the pellet was frozen at −80 °C, the latter being the protein matrix for further freeze-drying.

### 2.6 Attenuated Total Reflectance-Fourier Transform Infrared (ATR-FTIR) Spectroscopy

To assess the protein–polyphenol affinity in matrices, ATR-FTIR spectra were recorded on a Nicolet IS10 spectrophotometer equipped with a Smart Orbit ATR accessory (Thermo Scientific). A gold mirror was set up for the background spectra. The spectral zone of interest involving amide bonds, in particular, is magnified between 1800 and 1200 cm^–1^.

### 2.7 Supernatant Analysis after Protein–Polyphenol Matrix Formation

Both extraction and HPLC analyses were performed using the above-mentioned procedures, where the gradient, flow rate, and detection of polyphenols were specified (2.4). The chromatographic protocol involved a water (A)–CH_3_CN (B) mobile phase containing 0.1% (v/v) HCOOH.

### 2.8 Collection of Human Sera

Individual sera were collected from ten allergic patients at the University Hospital *Fundación Jiménez Díaz* (Madrid, Spain). All patients showed sensitivity to casein, with six showing sensitivity to both casein and β-lactoglobulin. The diagnosis of allergy was based on a history of symptoms triggered by cow milk consumption, with positive skin prick testing. Specific levels of IgE were assessed with ImmunoCAP (Thermo Fisher) using cow’s milk extract and pure proteins Bos d 5 and Bos d 8, following the manufacturer’s instructions.

### 2.9 Ethical Statement

This research has been approved by the *Fundación Jiménez Díaz* Ethics Committee (PIC 201-20-FJD, November 13, 2020), and the Bioethics and Biosafety Committee at the University of Extremadura (179/2021, December 20, 2021). All patients involved in this investigation agreed with the study’s objectives and signed an informed consent declaration. This study was conducted in agreement with the Declaration of Helsinki.

### 2.10 Evaluation of IgE Reactivity against Protein–Polyphenol Matrices

Indirect ELISA was employed to evaluate IgE reactivity of the protein–polyphenol complexes. A total of 0.5 μg of previously quantified protein, using Pierce Coomassie Plus Reagent (Thermo Fisher Scientific), was applied to the wells of 96-well Costar EIA plates (Corning, NY, USA) in phosphate buffer (pH 7.4) and incubated overnight at 4 °C to ensure proper plate coating. After blocking for 1 h with PBS–Tween20 0.1% (v/v) containing 3% (w/v) skim milk, diluted sera from the allergic patients were added to the plates and incubated for 2 h. Following three washes with PBS–Tween20 0.05% (v/v), a secondary mouse antihuman IgE antibody (ALK-Abelló) was applied at a 1:5000 dilution for 1 h. Then, goat-antimouse IgG (Bio-Rad, Hercules) at a 1:3000 dilution was added for 1 h. After the final wash, the ELISA was developed by adding 3,3′,5,5′-tetramethylbenzidine (TMB, Merck), and the optical density (OD) was measured at 650 nm after a 20 min color development reaction. Negative controls involved testing atopic patient serum and polyphenol matrix alone in parallel.

### 2.11 Statistical Analysis

Comparative statistics were conducted using Student’s *t-*test with significance given by *p* < 0.05. Data were analyzed using the SPSS 17.0 software for Windows (SPSS Inc.) and Sigmaplot 15.0 (SYSTAT Software). All measurements are presented with corresponding standard deviations.

### 2.12 Computational Modeling

#### 2.12.1 Methodology I: Receptor Modeling and Binding Pocket Scan

I-TASSER (Iterative Threading ASSEmbly Refinement)^24,25^ constitutes a widely used online server for protein structure prediction and function annotation. It generates 3D models of protein structures by identifying structural templates from the Protein Data Bank (PDB) through multiple threading alignments, followed by iterative template fragment assembly simulations. I-TASSER was chosen because it has been extensively used in characterizing the binding landscape of natural products to caseins in molecular docking campaigns (see the main text). According to this background, caseins α-S1, α-S2, β, and κ were modeled via the I-TASSER server with their FASTA sequences, obtained from UniProt (accession codes for *Bos taurus* types P02662, P02663, P02666, and P02668, respectively).^26^ The crystal structure of β-lactoglobulin was retrieved from the Protein Data Bank (PDB ID: 7ER3) and was subsequently stripped of its cocrystallized ligand, chloroquine.^27^ β-Lactoglobulin and casein models with the highest C-score were retained and progressed to Flare for protein preparation (normal calculation mode) for ionization tuning at pH = 7 and optimal small side-chain adjustments.^28^ Once the 3D structures were prepared, the proteins were scanned for potential binding pockets using Flare’s pocket detection module, which is based on the fpocket algorithm.^29^ Detected regions with a druggability score higher than 0.3 in each casein isoform were retained for independent docking runs. Lowering the recommended 0.5 to 0.3 allowed the inclusion of higher polar pockets found in other receptors, such as β-lactamases, HMG-CoA, or lysozymes.^30^ Detected pockets were considered on a qualitative basis, i.e., no further discussion about druggability score values was taken into account.

#### 2.12.2 Methodology II: Molecular Docking Calculations

Noncovalent docking campaigns were performed using Flare′s docking module, Lead Finder.^31,32^ Each isoform contains four profiled binding pockets, which were taken as docking spaces. A grid box of 10 Å centered in each region was built, where Lead Finder genetic algorithm exercises were carried out with the two polyphenols (normal mode). Owing to the explorative character of the present analysis, the Lead Finder Virtual Screening score (LF VSscore) was the scoring function selected as the binding quality feature.

## 3 Results and Discussion

### 3.1 Formation and Characterization of Protein-Bound Polyphenols

Matrices comprising cow milk proteins (either β-lactoglobulin or casein) and the aqueous extract obtained from lemon peel, as well as those involving such proteins and the pure polyphenols detected within the natural extract for comparative assessment, could be easily obtained and isolated by freeze-drying (see Supporting Information). These matrices were evaluated at different protein–polyphenol extract ratios (1:2, 1:1, and 2:1, respectively) and at room temperature, as a previous study showed little or no effect of temperature on matrix formation.^19^ Given the chemical heterogeneity of the resulting biomarkers, we chose an analytical approach combining Fourier Transform Infrared (FTIR) spectroscopy (ATR mode) and HPLC for monitoring. FTIR spectroscopy is the technique of choice to detect the changes in secondary structures associated with amide bands.^18,33^ Significant changes in shift and intensity are generally indicative of strong interactions, but they may not correlate with IgE binding responsiveness. Figure 1 shows the superimposed spectra of unmodified casein and its extracts with polyphenols from lemon peel at the aforementioned ratios.

**Figure 1:**
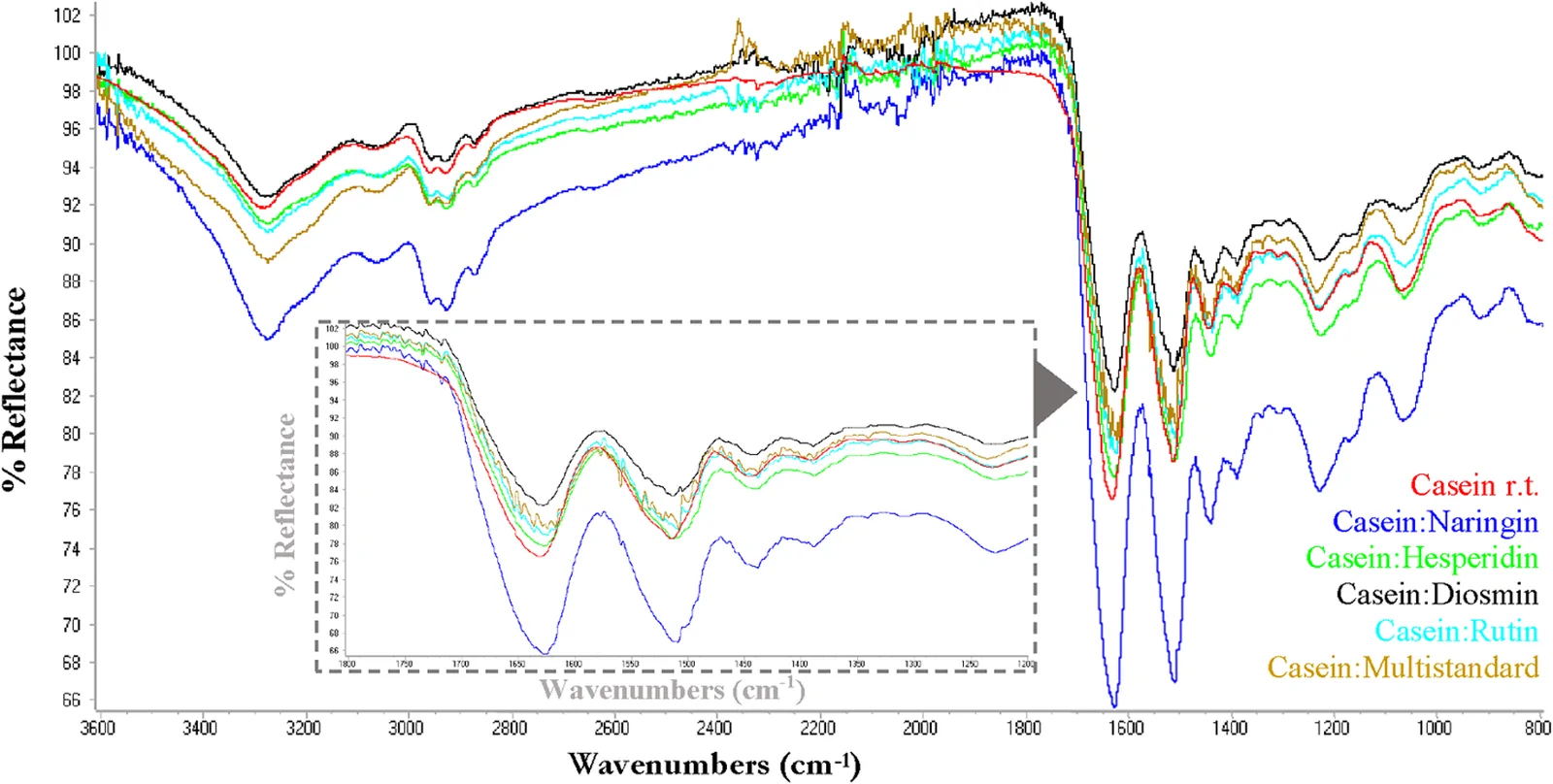
FTIR (ATR mode) spectra of native casein and matrices generated with polyphenols from lemon peels at room temperature. The inner inset shows an enlargement of the corresponding amide band.

There are variations in intensity corresponding to stretching C–H vibrations below 2900 cm^–1^, and most bands undergo changes in the fingerprint region (1350–800 cm^–1^), characteristic of skeletal vibrations, notably amide bands I and II lying in the range of 1800–1200 cm^–1^. The highest intensity was observed for the 2:1 casein-extract matrix, whereas similar spectra were obtained for the others and native casein, at least within the wavenumber region of interest. Table 1 lists the percentages of interactions quantified in terms of polyphenols present in the supernatant after matrix formation. Regardless of the ratio employed, hesperidine showed the highest value, which was nevertheless lower than that measured for this polyphenol in hen egg protein extracts.^34^ In general, not only for this *O*-glycoside, but also for other polyphenols, the extent of interaction was greater at a 2:1 ratio, which was taken as a reference for the subsequent preparation of casein-based formulations.

**Table 1:** Identification and Quantitative Analyses of Phenolic Compounds from Lemon Peel Extract (Supernatant) after Matrix Formation with Casein^a^,^c^

**polyphenol**	**concentration in extract (mg/g extract)** ^b^	**concentration in supernatant (mg/g extract)** ^b^	**interaction (%)**
**Ratio** 1:1			
**Naringin**	0.563 ± 0.038	1.479 ± 0.030*	-
**Hesperidin**	9.662 ± 0.087	7.612 ± 0.032*	21.2
**Eriocitrin**	2.627 ± 0.071	2.173 ± 0.009*	17.3
**Rutin**	0.130 ± 0.023	0.155 ± 0.010	-
**Diosmin**	0.115 ± 0.011	0.092 ± 0.005	20
**Ratio** 1:2			
**Naringin**	0.563 ± 0.038	1.223 ± 0.023*	-
**Hesperidin**	9.662 ± 0.087	6.924 ± 0.049*	28.3
**Eriocitrin**	2.627 ± 0.071	2.039 ± 0.001*	22.4
**Rutin**	0.130 ± 0.023	0.152 ± 0.006	-
**Diosmin**	0.115 ± 0.011	0.097 ± 0.006	15.6
**Ratio** 2:1			
**Naringin**	0.563 ± 0.038	1.195 ± 0.007*	-
**Hesperidin**	9.662 ± 0.087	6.403 ± 0.013*	33.7
**Eriocitrin**	2.627 ± 0.071	1.893 ± 0.043*	26.5
**Rutin**	0.130 ± 0.023	0.132 ± 0.009	-
**Diosmin**	0.115 ± 0.011	0.086 ± 0.006*	25.2

a All preparations were conducted at room temperature with quantification by HPLC analyses.

b Data are expressed as mean ± standard deviation.

c There were significant differences (*p* < 0.05) relative to the initial concentration in the extract.

It is worth pointing out that the abnormal data obtained for certain polyphenols exceed the concentration relative to the parent extract in the supernatant. This could be attributed to oxidative transformations, which have been reported for related compounds, such as the hesperidin–diosmin conversion through spontaneous dehydrogenation.^35^

To evaluate the possibility of an artifact caused by polyphenols in the extract (matrix effect) along with a synergistic effect, matrices involving casein and polyphenols were obtained alone and combined in a multistandard solution, and the concentration found in the peel extract doubled. These formulations were generated for the main polyphenols present: naringin, hesperidin, rutin, and diosmin, while expensive eriocitrin was ruled out. Again, superimposed FTIR spectra (Figure 2) showed changes in the band intensity while maintaining similar wavelength shifts. Relative to casein, the highest intensity was found for the casein–naringin matrix, which was almost negligible for diosmin.

**Figure 2:**
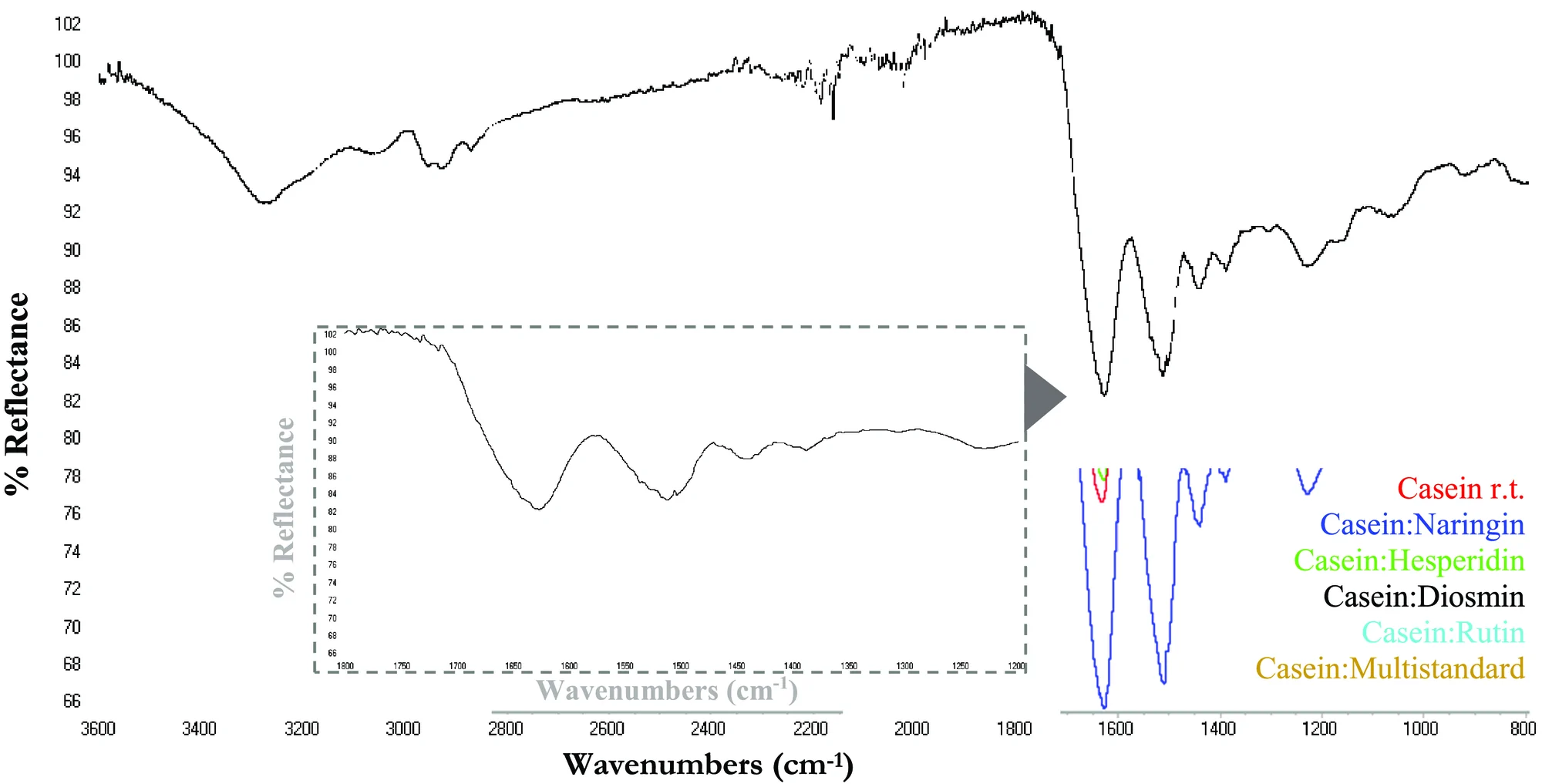
FTIR (ATR mode) spectra of casein and its matrices with neat polyphenols and a multistandard solution. The inner inset shows an enlargement of the corresponding amide bands I and II (1800–1200 cm^–1^).

Data collected in Table 2 indicate that for single polyphenols, the most significant interaction percentages were obtained for diosmin and rutin. Application of the above FTIR monitoring and chromatographic analysis to casein–polyphenol matrices (alone and present in multistandard solution) with double concentration of phenolic compounds gave rise to similar profiles (Figure S1 and Table S1), i.e., high-intensity peaks were observed for the casein–naringin complex, whereas from a quantitative standpoint, diosmin and rutin appear to interact with casein to a greater extent based on the amount of free polyphenol measured in the supernatant. Such concentrations were, however, slightly lower than those observed for the same polyphenols in the lemon peel extract.

**Table 2:** Quantitative Analysis of Polyphenols (Supernatants) after Matrix Formation^a^ ^,^ ^c^

**polyphenol**	**concentration in the starting solution** (mg/L)^b^		**concentration in supernatant** (mg/L)^b^	**interaction (%)**
**Naringin**	2.502 ± 0.067		2.497 ± 0.005*	0.2
**Hesperidin**	43.852 ± 0.043		7.920 ± 0.013*	81.9
**Diosmin**	0.463 ± 0.006		0.049 ± 0.002*	89.4
**Rutin**	0.548 ± 0.017		0.086 ± 0.007*	84.3
**Multistandard**	Naringin	2.599 ± 0.081	2.287 ± 0.026*	12.0
	Hesperidin	43.825 ± 0.034	22.334 ± 0.042*	49.0
	Diosmin	0.474 ± 0.012	0.095 ± 0.006*	79.9
	Rutin	0.570 ± 0.017	0.091 ± 0.003*	84.0

a Determined by HPLC analysis.

b Data are expressed as mean ± standard deviation.

c There were significant differences (*p* < 0.05) relative to the initial concentrations.

Identical protocols were extended to β-lactoglobulin, the other major allergen in patients allergic to cow’s milk, i.e., first assessing the combination of this protein with the aqueous extracts from lemon peels, which allowed us to unveil the specific interactions of the most abundant polyphenols with casein. Next, casein–polyphenol matrices were prepared, with each polyphenol alone, in a multistandard mixture, and with double concentration. Regarding raw lemon peel extracts, FTIR spectra showed greater changes in the band intensity for the 1:2 protein:extract combination than for the 1:1 and 2:1 ratios (Figure S2). HPLC quantification of complex formation, anew based on the remaining supernatant composition, showed the highest values for hesperidin and eriocitrin at 2:1 ratios, in line with the above results for casein (Table S2). When polyphenols are taken individually, spectral changes (Figure S3) mismatched those observed for casein, with rutin causing major changes in band intensity and naringin causing minor ones. Notably, the degree of interaction inferred from chromatographic analysis (Table S3) points to rutin and diosmin as ligands complexing to a greater extent, which is opposite to that found for the extract. With double concentration, the multistandard mixture led to the greatest change in the IR band intensity, followed in sequential order by diosmin, naringin, and rutin (Figure S4), whereas quantitative interactions established by HPLC mirrored well the results shown in Table S3, i.e., rutin and diosmin reached the highest percentages (Table S4).

### 3.2 IgE Responsiveness of Protein–Polyphenol Matrices in Human Sera

Hypoallergenic formulas suitable for OIT should show less (or at least the same) ability to bind specific IgE from serum, aiming to reduce the risk of adverse reactions when the patient is exposed to the allergen–polyphenol matrix. Early introduction of OIT, particularly in children, is invariably problematic, and side reactions may occur if other symptoms or minor pathologies (like eczema) are present.^36,37^ For some allergies, a common formulation lies in thermal inactivation of the allergen, thereby decreasing IgE reactivity,^38,39^ whereas chemical modifications are less exploited. In this context, the latter accounts for the present bottom-up elaboration of protein chimeras based on lemon polyphenols. To validate our approach, immunoassays were conducted on human serum taken from ten cow’s milk allergic patients (age range: 1–50 years). All had experienced severe and, in some cases, life-threatening symptoms after cow milk ingestion (Table 3). All patients showed serum-specific IgE (s-IgE) to casein (Bos d 8) and six to both casein and β-lactoglobulin (Bos d 5). Results of the immunoassays involving casein- and β-lactoglobulin-modified matrices are detailed in Figures 3 and 4, respectively.

**Figure 3:**
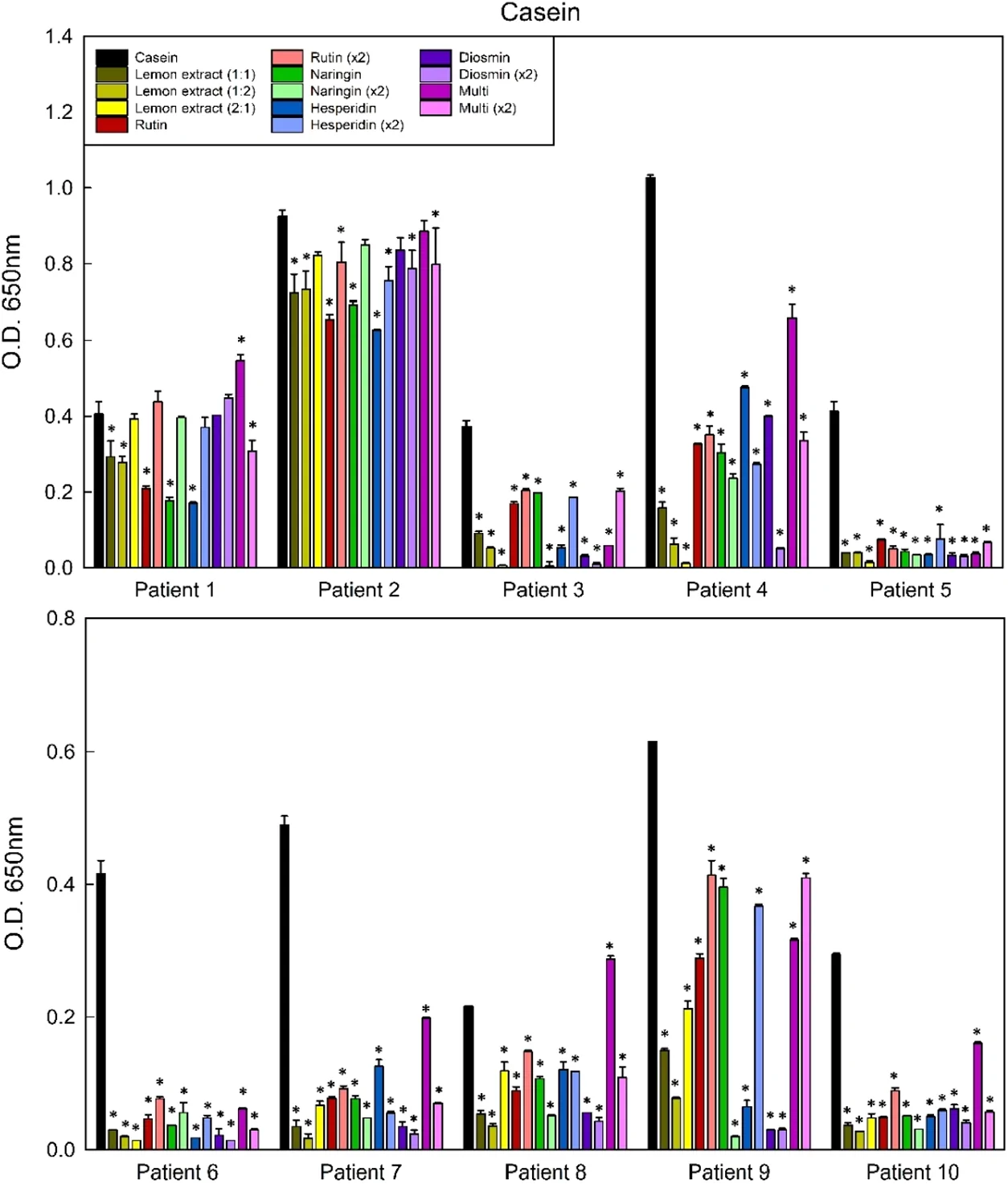
Indirect ELISA using sera from ten patients allergic to casein, using the native protein (black) and different protein–polyphenol matrices. Statistically significant differences between the matrix and native protein are indicated with an asterisk (*p* < 0.05).

**Figure 4:**
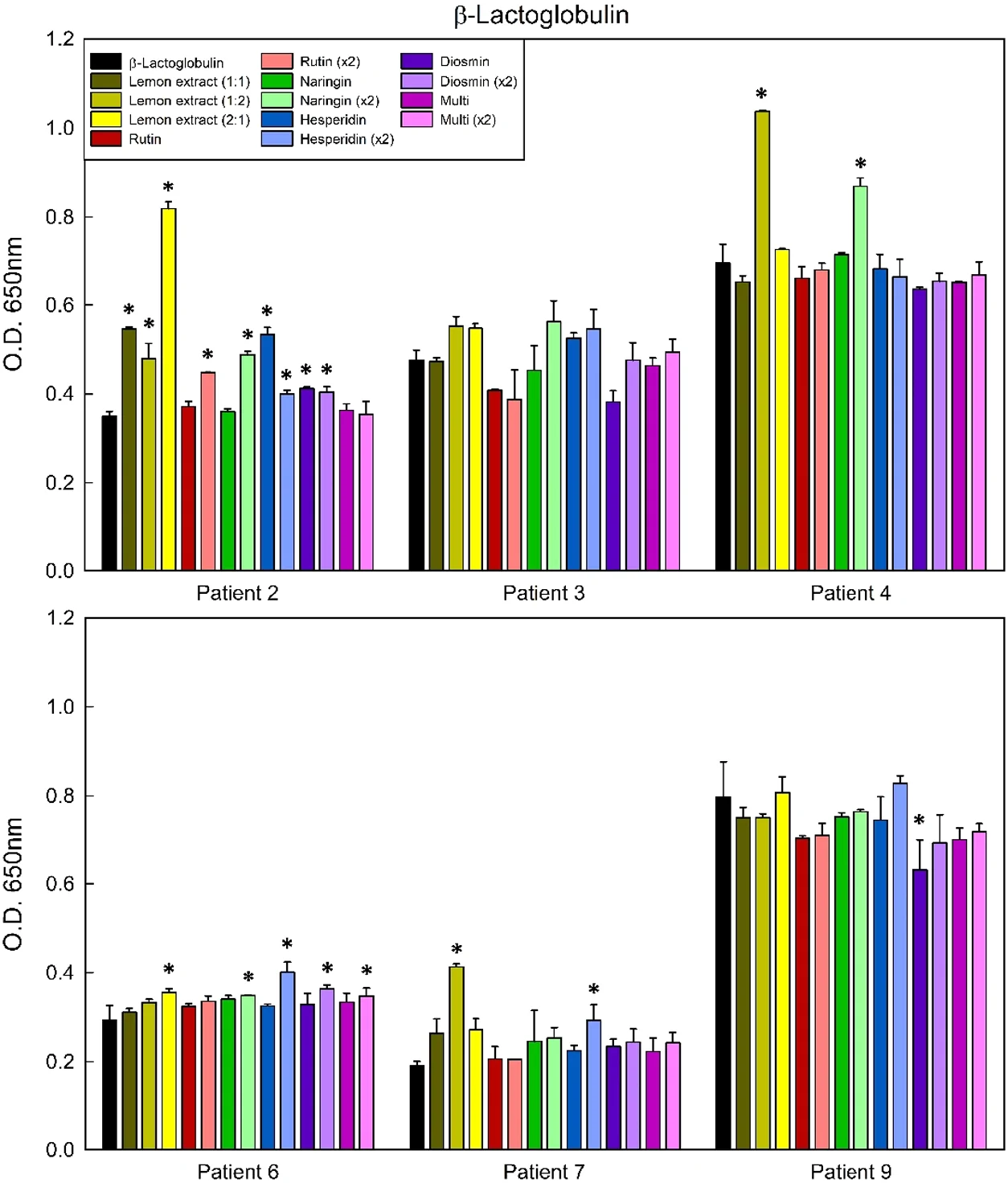
Indirect ELISA using sera from six patients allergic to β-lactoglobulin, using the native protein (black) and different protein–polyphenol matrices. Statistically significant differences between the matrix and native proteins are indicated by an asterisk (*p* < 0.05).

**Table 3:** Clinical Characteristics and Records of Patients Allergic to Cow’s Milk^a^

**number of patients**	**age**	**sex**	**total IgE (kU/L)**	**allergic reaction**	**IgE to cow’s milk (kU/L)**	**IgE to Bos d 5 (kU/L)**	**IgE to Bos d 8 (kU/L)**
**1**	20	F	622	Anaphylaxis	5.27	0,77	4.79
**2**	23	F	433	Anaphylaxis	>100	10.1	>100
**3**	50	F	380	NA	0.65	0.95	0.84
**4**	20	M	573	NA	>100	54.3	99
**5**	1	F	67,5	Syst.	4.66	0	6.08
**6**	1	M	2888	OAS	20.7	19.2	10.9
**7**	27	M	NA	Anaphylaxis	NA	NA	NA
**8**	18	F	NA	Anaphylaxis	11.9	2.56	5.48
**9**	21	F	554	Anaphylaxis	>100	>100	>100
**10**	20	F	546	Syst.	47.6	0.79	71

a F: female, M: male, OAS: oral allergy syndrome, Syst.: systemic reaction, and NA: not available.

Figure 3 shows that both lemon extract and polyphenols (rutin, naringin, hesperidin, and diosmin), taken individually or in a multistandard solution, combined with casein, generally exhibit reduced IgE binding across the patients involved in this study. Note that polyphenols were used at the concentration measured in the extract and at double (2×) the concentration.

Eight of them (patients 3–10) showed little selectivity toward a specific matrix within the detection limits provided by indirect ELISA (see Materials and Methods and SI). For patients 1 and 2, the IgE reactivity decreased significantly against rutin, naringin, and hesperidin, in particular when such polyphenols were present at the same concentration as found in the raw extract. This observation may suggest differences regardless of the patient’s IgE levels: patient 2 (Bos d 8 sIgE > 100 kU/L) recognized all the matrices, whereas patient 9 with identical IgE values showed little reactivity to some hybrids, such as casein–diosmin.

However, β-lactoglobulin–polyphenol matrices (Figure 4) did not show an IgE reactivity decrease related to hypoallergenic properties. While the results do not invalidate their use for OIT, as polyphenols can trigger numerous cascade reactions like those occurring during desensitization,^12,13,14,17,40^ it provides evidence that molecular screening of protein–ligand formulations should precede any OIT treatment. Having different polyphenols at the patient’s disposal, depending on the ability to recognize a given epitope, will increase selectivity and safety. Casein and β-lactoglobulin have different physicochemical properties, which may result in different protein–polyphenol interactions. In the case of casein, these could conceal relevant IgE epitopes, whereas β-lactoglobulin binds to nonepitopic regions. To analyze these differences in the context of protein–ligand binding, we performed docking analysis.

### 3.3 Molecular Modeling of Casein–Polyphenol Interactions

*How did casein*–*polyphenol matrices become hypoallergenic?* Fine-tuning casein is challenging due to its variable isomorphism relative to other food proteins, thereby making it difficult to disentangle how a ligand perturbs the native pocket of several active conformations. Nonetheless, our above observations show that polyphenols led to a significant reduction in IgE reactivity in patients sensitized to bovine milk casein. The latter suggests that one or more allergenic casein isoforms are modified because of the heterogeneous profiles present in the patient cohort.

Caseins (α-S1, α-S2, β, and κ) are random coil-enriched phosphoproteins that account for about 80% of the total protein content in bovine milk. Their high proline content disrupts ordered secondary structures, resulting in highly flexible “rheomorphic” conformations, particularly β-casein, which exhibits notable surface activity and amphiphilic behavior. Casein’s highly flexible conformational ensembles lead them to participate in reversible self-association processes as micellar organization.^41^ These structural features enable caseins to bind various bioactive molecules, including phenolic compounds, through noncovalent interactions (e.g., hydrogen bonding, hydrophobic forces, van der Waals) or, under specific conditions, covalent bonding. Such interactions can modify the protein’s structural and functional properties—such as solubility, emulsification, foaming, self-aggregation, and antioxidant capacity—supporting the use of phenolic compounds as natural stabilizers in protein-rich food systems and functional food development.

A plethora of computational studies employing molecular docking techniques has been dedicated to exploring the interactions between casein proteins and a wide array of naturally occurring bioactive compounds. Prospective binders studied includes compounds such as α-tocopherol,^42^ apigenin,^43,44^ caffeic acid,^45^ catechin,^46^ chrysin,^43^ curcumin,^47^ delphinidin 3-*O*-glucoside,^48^ ellagic acid,^49^ epicatechin,^46,50^ epicatechin gallate,^50^ epigallocatechin,^46,50,51^ epigallocatechin gallate,^44,46,50,51^ gallic acid,^45,49,51^ genistein,^47^ glucomannan,^52^ kaempferol,^49^ luteolin,^43,44^ *m*-hydroxybenzoic acid,^49^ myricetin,^49^ *p*-coumaric acid,^53^ *o*-hydroxybenzoic acid,^49^ *p*-hydroxybenzoic acid,^49^ quercetin,^44,49,51^ quercetin 3-glucoside,^45^ quercitrin,^51^ resveratrol,^47^ rutin,^54^ tannic acid,^49,51^ *trans*-ferulic acid,^55^ vitamin B12,^56^ or xylitol.^57^ In general, these studies strengthen experimental research related to the modifications caused by such natural compounds on the structure and functional properties of caseins.

The present work aims to explore the possible binding modalities of polyphenols toward different casein subtypes to provide an explanation for the reduced IgE reactivity of casein–polyphenol matrices. Due to the reversible self-aggregation mechanism of casein micelles, our approach is intended as an exploratory, bottom-up framework to characterize potential interaction hotspots between phenolic compounds and individual casein isoforms.^x2^ Therefore, the objective is to characterize the nature and specificity of these interactions, contributing to a deeper understanding of how structural diversity among polyphenols may influence their affinity for κ-, α-, and β-isomorphs. This approach seeks to provide molecular-level insights that complement existing empirical data and support future applications in food science and functional protein systems.

Our methodology first involved receptor modeling and ligand binding using I-TASSER (Iterative Threading ASSEmbly Refinement),^58,59^ a widely employed online server for protein structure prediction and function annotation. Caseins α-S1, α-S2, β, and κ were modeled via the I-TASSER server with their FASTA sequences, obtained from UniProt.^26^ It should be noted that owing to the exploratory character of this scouting analysis, detected pockets should largely be regarded in qualitative terms, i.e., no druggabbility scores have been considered. Noncovalent docking campaigns were performed using Flare′s docking module, Lead Finder^31,32^ (see Supporting Information, Section S2, for a detailed description of the above-mentioned protocol).

α-S1 type casein was predicted as a rod-like structure with a helical-like secondary structure in good agreement with published data highlighting both secondary and tertiary structures (Figure 5).^48,56^ Three putative binding pockets were detected: two in the protein terminals (as1_bp2 and as1_bp3) and the last one, higher in score, close to the global turn (Table 4).

**Figure 5:**
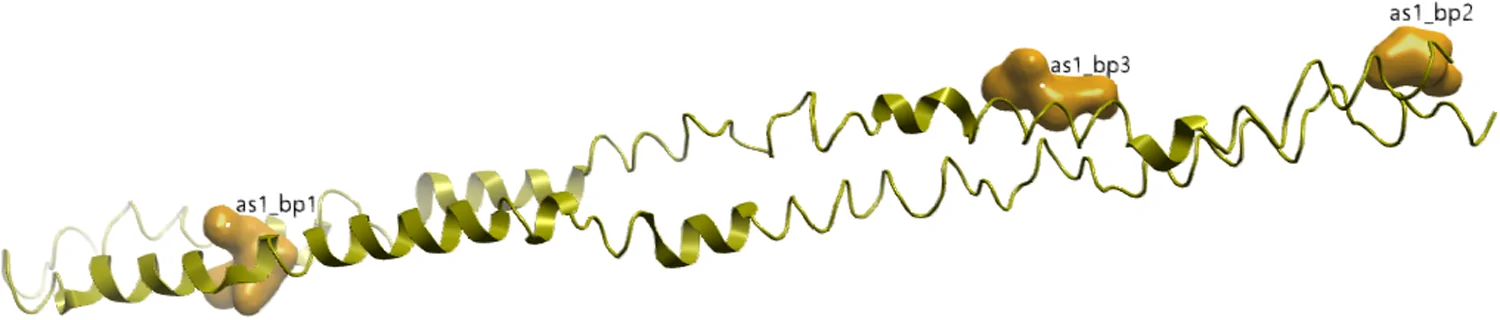
Predicted structure of α-S1 casein with the detected binding pockets as1_bp1 to bp3.

**Table 4:** Binding Pocket Features for α-S1 Binding Pocket Scouting Runs

**isoform**	**binding pocket**	**druggability score**	**residues**	**epitope ID (IEDB)**
**α-S1**	as1_bp1	0.488	EIVPNSVEQ (85–93)	11704
			LEIVPNSAEER (124–134)	35530
	as1_bp2	0.348	MKLLILTCLVA (1–11)	57509
			EKTTMPLW (207–214)	
	as1_bp3	0.317	QGLPQEVLNENL (24–35)	190448
			YYVPLGTQYTDAPSFSDIP (180–198)	115189

Molecular docking results revealed that both diosmin and rutin exhibit favorable binding affinities toward the three identified binding pockets (Table 5). Diosmin binds competitively to all three binding pockets (bp1–bp3) of α-S1 casein. Its strongest interaction occurs in bp1 when diosmin has no charge, with an LF VSscore of −11.11 kcal/mol (Figure 6). This high binding affinity appears to be driven by the larger number of hydrophobic contacts formed with residues such as V87, P88, E92, A131, and L135, in addition to key H-bonds. In comparison, its binding affinity decreases in bp2 and bp3 when the diosmin bicyclic core OH is deprotonated, with scores of −9.93 kcal/mol and −10.95 kcal/mol, respectively. Rutin shows preferential binding to bp1 and bp2 of α-S1 casein, particularly in its −2 ionization state, where it achieves its most favorable docking energies (−9.56 kcal/mol in bp1 and −9.73 kcal/mol in bp2). The Bp1-enhanced interaction is likely attributed to the higher number of hydrophobic contacts, reflected by a 0.488 druggability score value, suggesting a favorable nonpolar environment for rutin’s aromatic rings. In bp2, although hydrophobic interactions are present, a critical π–π stacking interaction with residue W214 appears to significantly stabilize the complex, making bp2 competitive with bp1 in terms of binding strength (Figure 6). Moreover, the three binding pockets cover totally or partially different IgE-binding epitopes in α-S1 casein, according to the IEDB database (iedb.org), which would explain the decrease in IgE reactivity of polyphenol-bound α-S1 casein.

**Figure 6:**
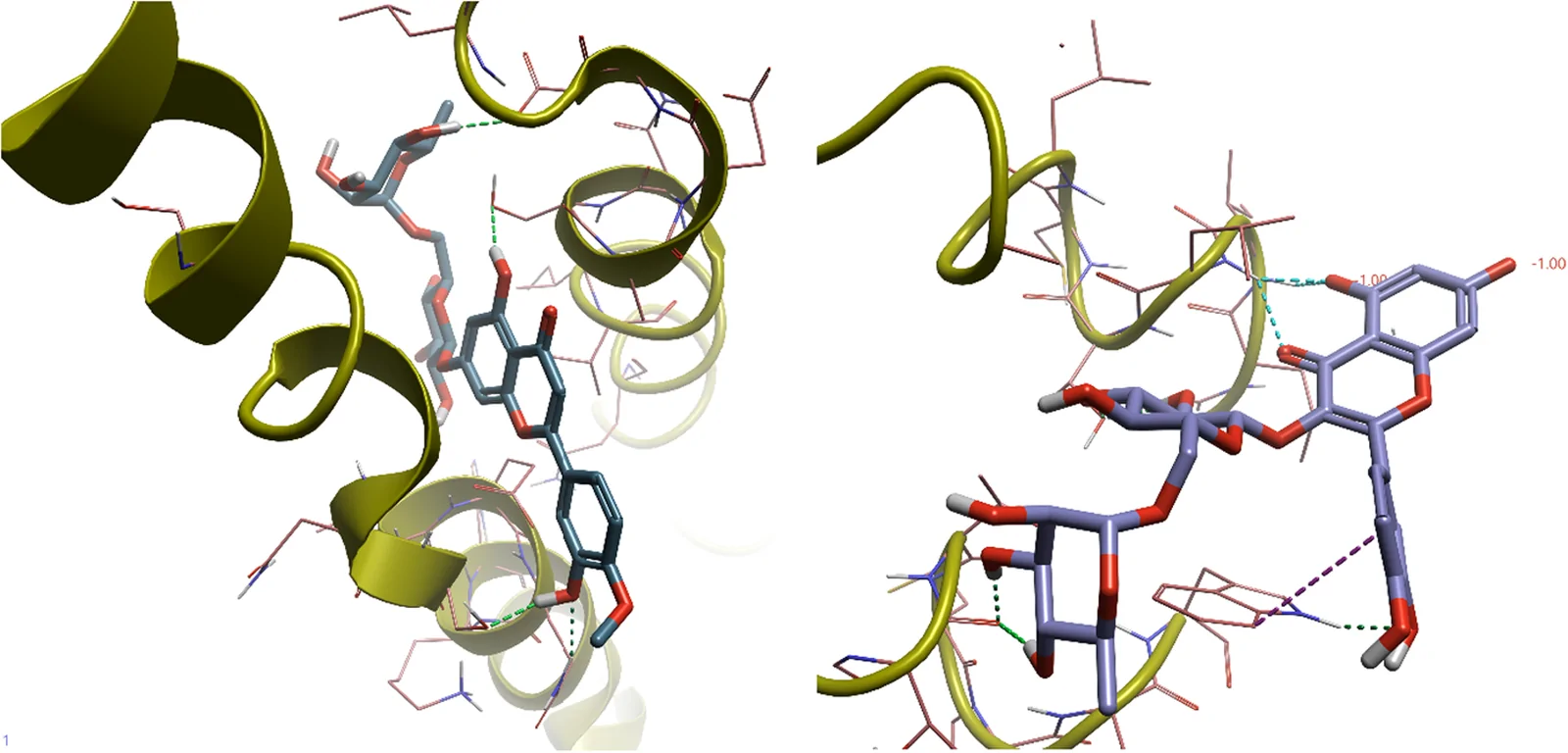
Binding topologies of diosmin (Table 5, entry 1, left-hand side) and rutin (entry 8, right-hand side) to α-S1 casein.

**Table 5:** Binding Topologies of Charged Diosmin and Rutin to α-S1 Binding Pockets

**entry**	**isoform**	**binding pocket**	**molecule**	**charge**	**LF VSscore** ^a^	**interactions**
**1**	**α-S1**	as1_bp1	Diosmin	0	–11.11	S90, Q93, S130, E133 (hb)
						V87, P88, E92, A131, L135 (hc)
**2**				–1	–9.487	E85, E92, E133, R134, M138 (hb)
						R134 (hc)
						R134 (cπ)
**3**			Rutin	–1	–8.95	S90, E92, Q93, S130, R134 (hb)
						V87, P88, A131 (hb)
**4**						R134 (cπ)
				–2	–9.56	I86, E92, Q93, S130, E133 (hb)
						V87, R92, I126, V127, L135 (hc)
**5**		as1_bp2	Diosmin	0	–8.94	T7, V10, N205, T209 (hb)
						L4, A11, T210, W214 (hc)
						W214 (ππ)
**6**				–1	–9.93	T7, V12, R16 (hb)
						C8, L9, V10 (hc)
**7**			Rutin	–1	–7.77	T7, L9, V12, L14, A15, R16 (hb)
						C8, L9, V10 (hc)
**8**				–2	–9.73	I5, L6, T7, T209, W214 (hb)
						L4, L6 (hc)
						W214 (ππ)
**9**		as1_bp3	Diosmin	0	–9.69	I21, N32, N34 (hb)
						P27, F39, T189, F194 (hc)
**10**				–1	–10.95	L26, N32, N34, T186 (hb)
						P27, N34, T189, F194 (hc)
						F194 (ππ)
**11**			Rutin	–1	–7.93	Y188, D196 (hb)
						P20, I21, Q28 (hc)
**12**				–2	–8.59	H23, Q24, Y188, D196 (hb)
						Q28 (hc)

a LF VSscores are given in kcal/mol.

Similarly to the α-S1 casein isoform, I-TASSER also predicted for α-S2 an elongated structure with a helical-like secondary structure (Figure 7). The lead binding pocket as2_bp1 (druggability score, 0.828) was found close to pockets as2_bp2 and as2_bp3 with lower scores (Table 6). All three binding pockets are located near the N- and C-terminal regions of the α-S2 casein structure, suggesting spatial clustering of potential ligand-binding sites toward such distal positions of the protein.

**Figure 7:**
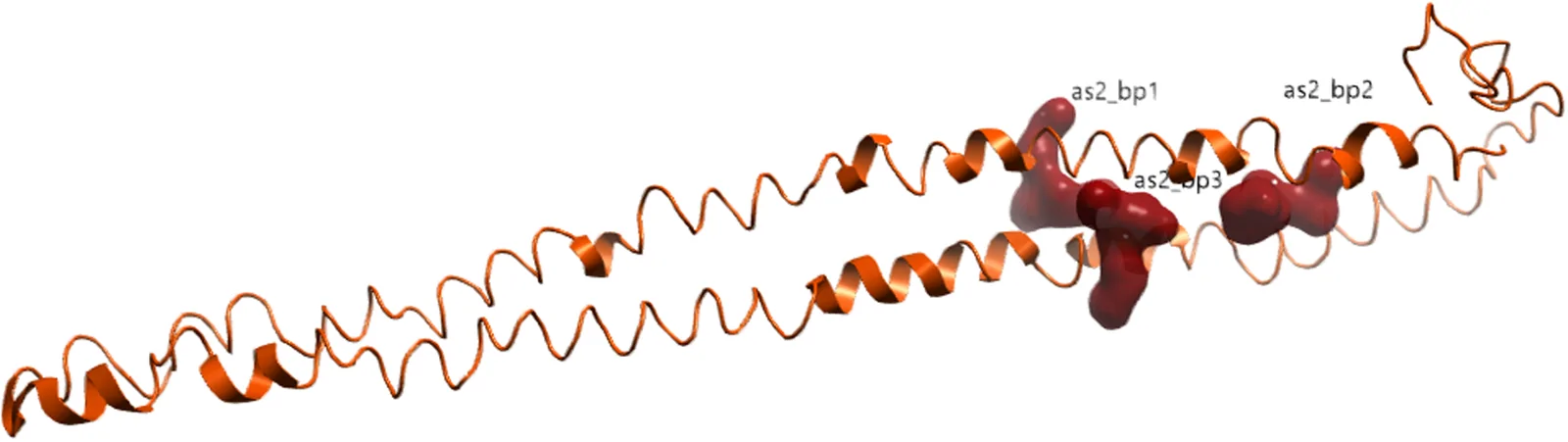
Predicted structure of α-S2 casein with the detected binding pockets as2_bp1 to bp3.

**Table 6:** Binding Features for α-S2 Binding Pocket Scouting Runs

**isoform**	**binding pocket**	**druggability score**	**residues**	**epitope ID (IEDB)**
**α-S2**	as2_bp1	0.828	ENLCSTFCKEVVRNANE (48–64)	115395, 115295
			QKFALPQYLKTVYQH (187–201)	2248466
	as2_bp2	0.539	ETYKQEKNMAINPSKE (33–48)	78269
			HQKAMKPWIQPKTKVI (201–216)	78189
	as2_bp3	0.371	NPSKENLCSTFCKE (44–57)	115295, 115399
			LPQYLKTVYQHQKAM (191–205)	2248466, 2246188

Topological binding exploration by molecular docking indicates that both diosmin and rutin bind with the three identified pockets of α-S2 casein (Table 7). Rutin shows preferential binding to bp2 and bp3, with bp3 emerging as the most favorable site (charge −2), achieving a LF VSscore of −10.35 kcal/mol. This higher affinity appears to be driven by an extensive H-bonding network involving residues K47, L50, T53, K56, and H201, in combination with hydrophobic contacts with C55, K56, and H201. On the other hand, diosmin exhibits a comparative inclination to bind all three pockets, with bp3 (charge 0) showing a higher binding score (−10.21 kcal/mol). Critical H-bonds with residues L50, T53, C55, K56, A190, K196, and T197, as well as hydrophobic contacts with L50, C51, C55, and T197, describe the diosmin binding profile. These results suggest that the enhanced hydrogen-bonding capacity of the ligand–pocket interactions in bp2 and bp3 plays a pivotal role in stabilizing both molecules within α-S2 casein, surpassing the interactions observed in bp1 (Figure 8). As in α-S1, the three binding pockets of α-S2 casein contain IgE-binding epitopes according to the IEDB database (iedb.org), accounting for the hypoallergenicity of polyphenol-bound α-S2 casein.

**Figure 8:**
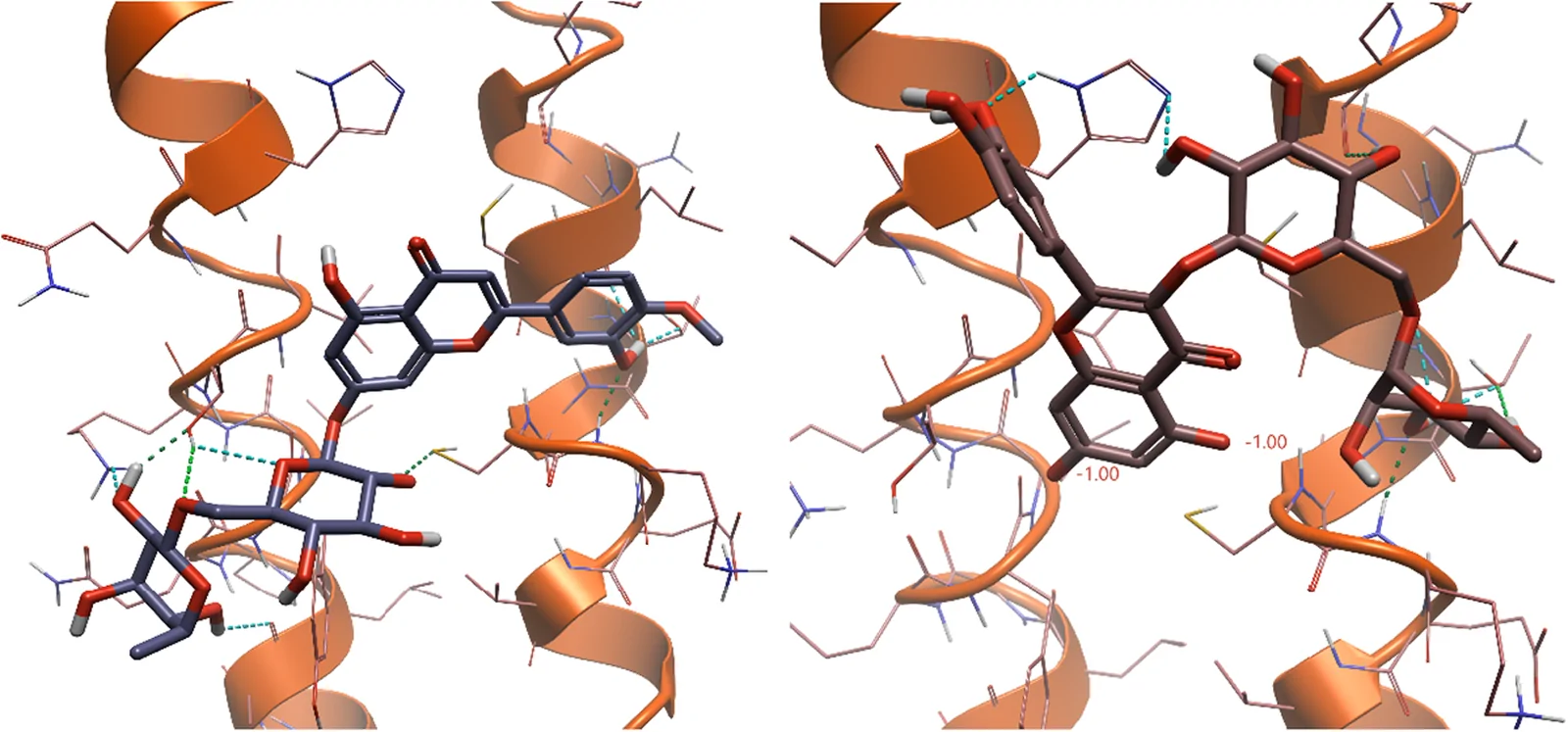
Binding topologies of diosmin (entry 9, left-hand side) and rutin (entry 12, right-hand side) to α-S2 casein.

**Table 7:** Binding Topologies of Charged Diosmin and Rutin to α-S2 Binding Pockets

**entry**	**isoform**	**binding pocket**	**molecule**	**charge**	**LF VSscore** ^a^	**interactions**
1	**α-S2**	as2_bp1	Diosmin	0	–9.49	C51, T53, C55, K56, Y194 (hb)
						C55, K56 (hc)
						Y194 (ππ)
2				–1	–9.62	C51, T53, C55, K56 (hb)
						C51 (hc)
3			Rutin	–1	–7.30	P192, L195 (hb)
						L191, P192 (hc)
						R185 (sb)
4				–2	–7.38	R185, P192 (hb)
						K188, L191, P192 (hc)
						R185 (sb)
5		as2_bp2	Diosmin	0	–9.95	A42, K47, Q200, A204, W208 (hb)
						M41, N44 (hc)
						H201 (ππ)
6				–1	–9.49	A42, I43, Q200 (hb)
						M41, N44, A204, M205 (hc)
						H201 (ππ)
7			Rutin	–1	–8.66	Q200, H201, A204, K203, W208 (hb)
						H201, A204, M205 (hc)
						H201 (ππ)
8				–2	–9.50	A42, M205, W208, Q210 (hb)
						A42, K47, I209 (hc)
						H201 (ππ)
9		as2_bp3	Diosmin	0	–10.21	L50, T53, C55, K56, A190, K196, T197 (hb)
						L50, C51, C55, T197 (hc)
10				–1	–9.91	L50, A190, Y194, K196, T197 (hb)
						C55, K56 (hc)
11			Rutin	–1	–9.26	L50, T53, C55, K56, T197 (hb)
						L50, T197, V198 (hc)
12				–2	–10.35	K47, L50, T53, K56, H201 (hb)
						C55, K56, H201 (hc)

a LF VSscores are given in kcal/mol.

β-Casein modeling successfully gave rise to a globular three-dimensional architecture, in agreement with previous structural descriptions reviewed in the literature (Figure 9).^43–45,47,53,55,56^ Two putative binding pockets were identified, both located within the interior of the structure, suggesting that ligand accessibility may depend on either local conformational flexibility or transient cavity exposure (Table 8).

**Figure 9:**
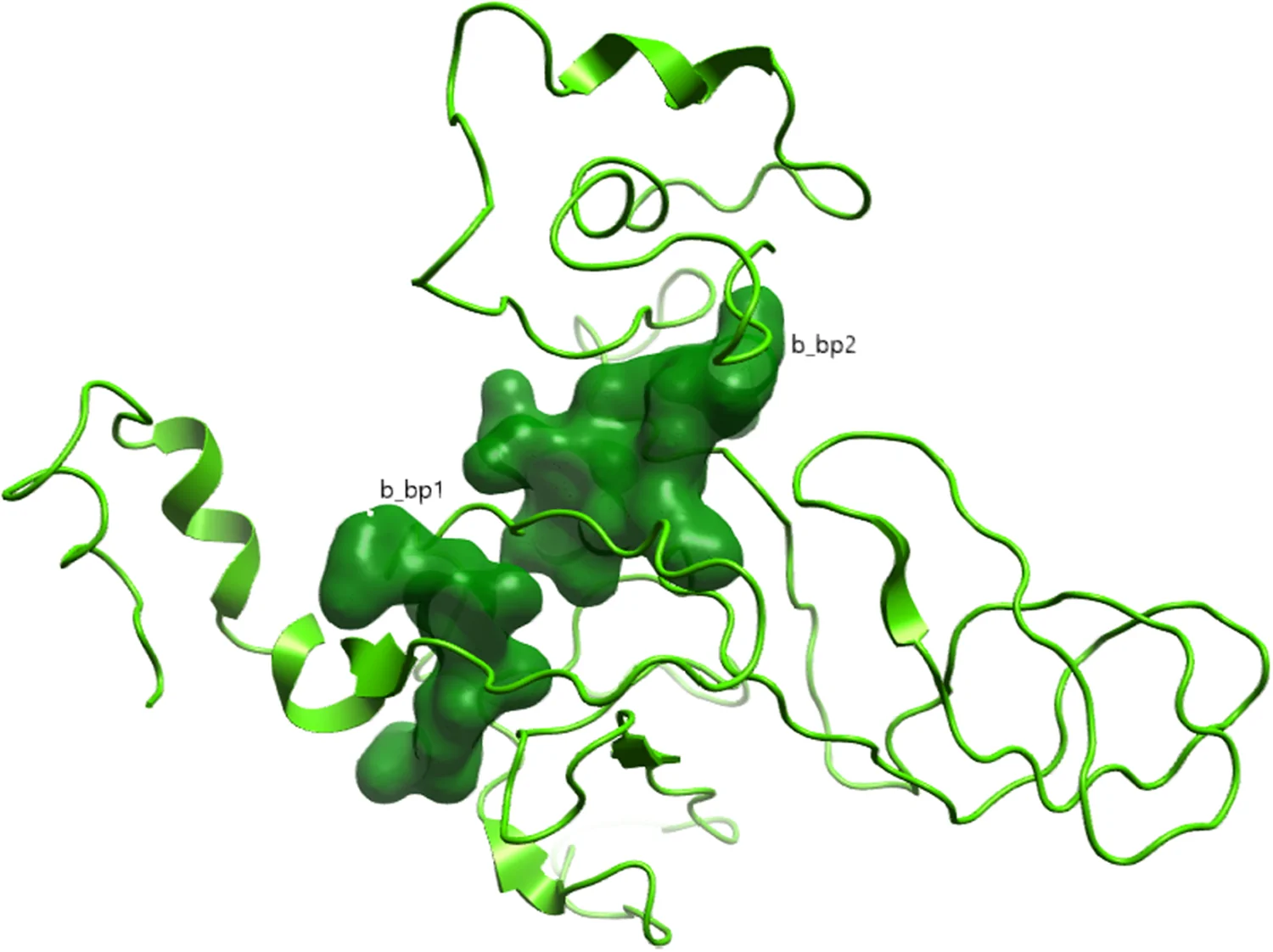
Predicted structure of β casein with the detected binding pockets b_bp1 to bp2.

**Table 8:** Binding Features of β-Binding Pocket Scouting Runs

**isoform**	**binding pocket**	**druggability Score**	**residues**	**epitope ID (IEDB)**
**β**	b_bp1	0.596	VPGEIVESLS (23–32)	115219,115408, 115466
			KKIEKFQSEEQ (43–53)	78259
			PF(66–67)	115895
			TPVVVPP (95–101)	115931
	b_bp2	0.350	SEESITRINKKIEK (34–47)	115538
			PFLQPE (101–106)	71789
			FT (134–135)	115906
			LPPTVMFPPQSVLSLS (166–182)	78287

It is noteworthy that both diosmin and rutin bind to β-casein with greater affinity than to the α-casein isoforms, a trend attributable to the globular nature of β-casein and the deeply buried position of its two identified binding pockets (Figure 10 and Table 9). This structural configuration appears to promote enhanced ligand–receptor interactions (in terms of stability), as reflected in the docking scores. Both ligands show a marked preference for bp2, regardless of their ionization state. Diosmin, in its neutral form, reached an LF VSscore of −14.89 kcal/mol, while rutin (charge −2) scored −13.43 kcal/mol. These interactions are not only stabilized by an extended H-bonding network (involving residues N42, K43, K44, E46, F102, Q104, E106, S181, L166, Y208, P221), but also by π–π stacking interactions with F134 and, in the case of diosmin, a salt bridge with K43. These residues are included in the corresponding binding pockets bp_1 and bp_2 of β-casein, which are also described as IgE-binding epitopes in IEDB (iedb.org).

**Figure 10:**
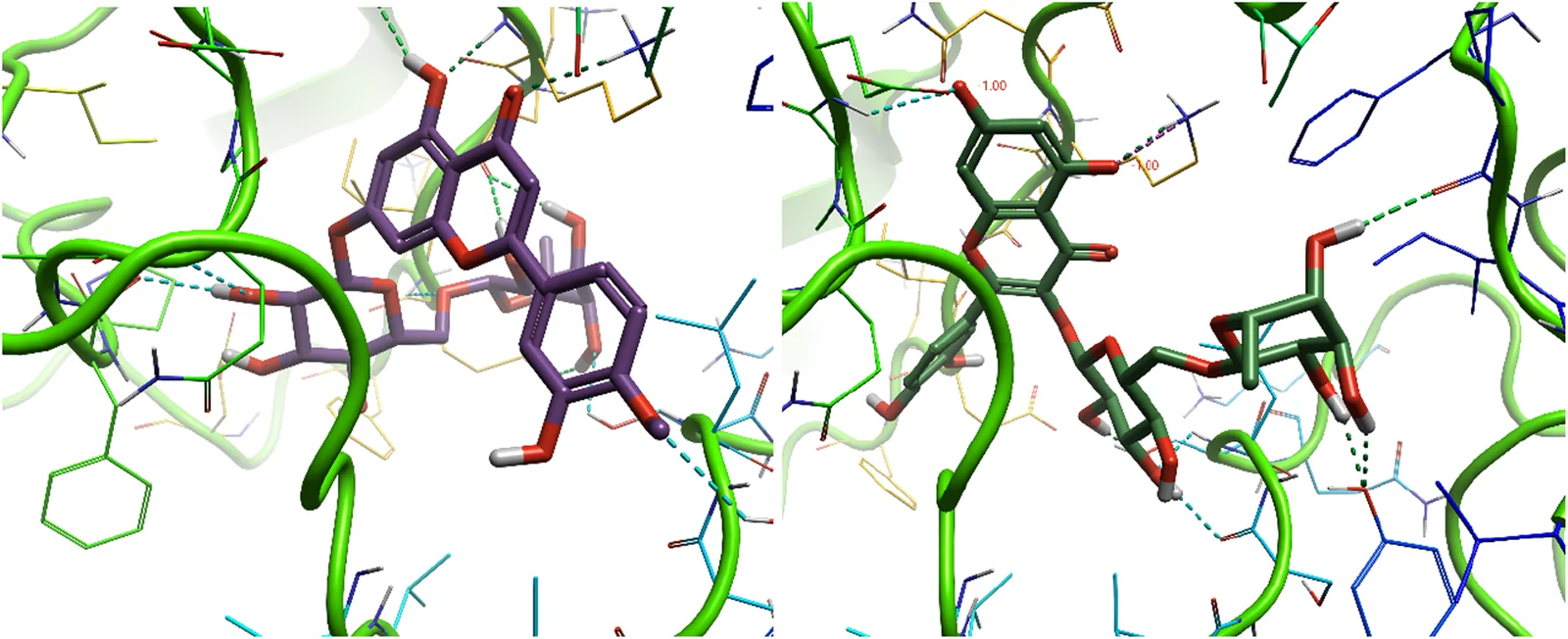
Binding topologies of diosmin (entry 5, left-hand side) and rutin (entry 8, right-hand side) to α-S2 casein.

**Table 9:** Binding Topologies of Charged Diosmin and Rutin to β-Binding Pockets

**entry**	**isoform**	**binding pocket**	**molecule**	**charge**	**LF VSscore** ^a^	**interactions**
1	**β**	b_bp1	Diosmin	0	–11.15	L31, S33, F67, Q69 (hb)
						V28, P66, F67, V99 (hc)
2				–1	–10.38	I27, S33, R40, L73 (hb)
						F67, Q71, L73 (hc)
						F67 (ππ)
3			Rutin	–1	–9.96	G25, E26, I27, E29 F67, T95 (hb)
						T95, V99 (hc)
4				–2	–9.74	G25, E26, E29, L31, S33, F67, A68 (hb)
						L31, F67 (hc)
5		b_bp2	Diosmin	0	–14.89	N42, K43, K44, E46, F102, Q104, S181, L166,
						Y208 (hb)
						K43, K44, Q104, P105 (hc)
				–1	–14.16	N42, K43, D62, Y75, E106, V107, Q156 (hb)
6						I41, N42, E106, M108, V131, F134 (hc)
						F134 (ππ)
7			Rutin	–1	–13.22	N42, K44, Q104, E106, S181, Q182, K184 (hb)
						K43, F134, L180 (hc)
						F134 (ππ)
				–2	–13.43	K43, E46, E106, S179, S181, Y208, P221 (hb)
8						K43, I45, Q104, P105, F134, L166, P168 (hc)
						K43(sb)

a LF VSscores are given in kcal/mol.

Concerning the last protein isoform, κ-casein modeling yielded a globular 3D-structure, characterized by a prominent helical domain, and consistent with structural features previously reported (Figure 11).^52,57^ A single binding pocket was identified, exhibiting a druggability score close to 1, indicating a highly druggable, well-defined cavity (Table 10). This site was, therefore, selected for subsequent molecular docking, as it likely represents the most pharmacologically relevant binding region within the protein.

**Figure 11:**
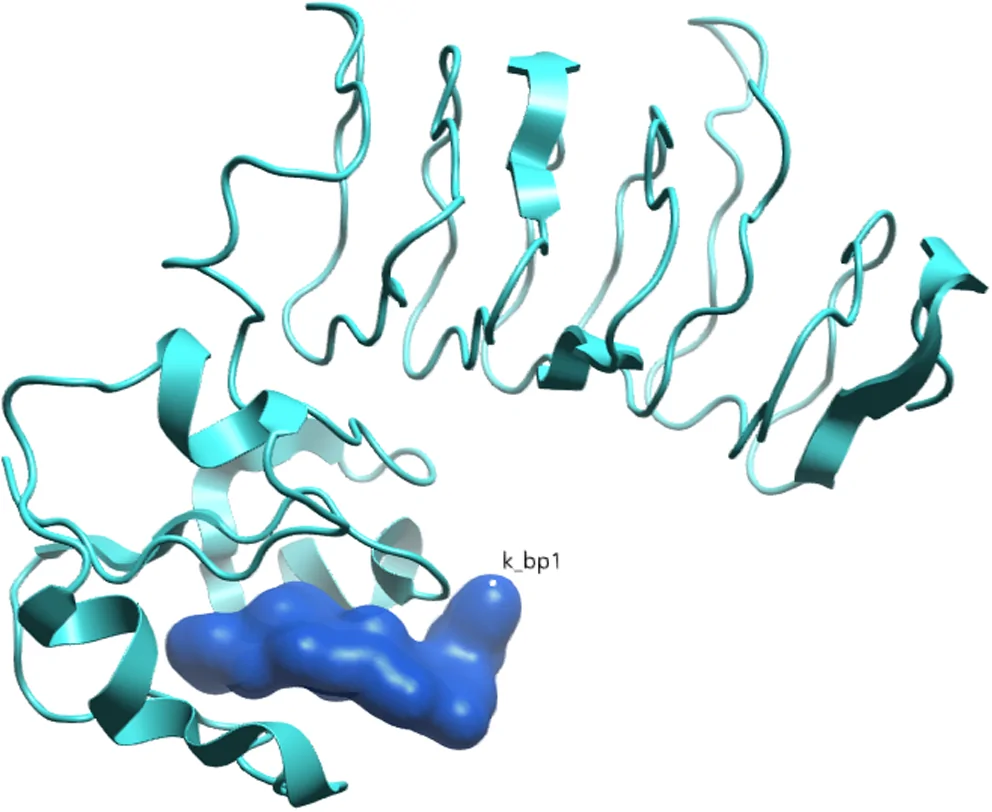
Predicted structure of κ casein with the detected binding pocket k_bp1.

**Table 10:** Binding Features for κ-Binding Pocket Scouting Runs

**isoform**	**binding pocket**	**druggability Score**	**residues**	**epitope ID (IEDB)**
**κ**	k_bp1	0.937	RCEKDERFFSDKIAKYIPIQYVLSRYPSYGLNYYQ (31–65)	78267

Finally, single binding pocket κ-casein exhibits the highest LF VSscores for diosmin, particularly in its −1 ionization state, with a LF VSscore of −16.99 kcal/mol, making it the most favorable binding across all casein isoforms analyzed (Figure 12 and Table 11). This high binding strength can be attributed to a dense H-bonding network involving residues Q24, Q28, R31, E33, K34, D35, E36, Y51, and R55, along with substantial hydrophobic contacts, including F6, R31, I47, Y51, V52, Y56, and L61. Rutin displayed competitive binding among charged species, with scores of −10.11 kcal/mol (charge −1) and −10.37 kcal/mol (charge −2). Nevertheless, residues Q26, C32, F39, and D41 are involved in both rutin binding topologies, with limited hydrophobic contacts. The only binding pocket in κ-casein also contains an IgE-binding epitope (78267, containing residues 46–64); thus, the polyphenol interaction modifies key residues involved in IgE binding, leading to potential hypoallergenicity.

**Figure 12:**
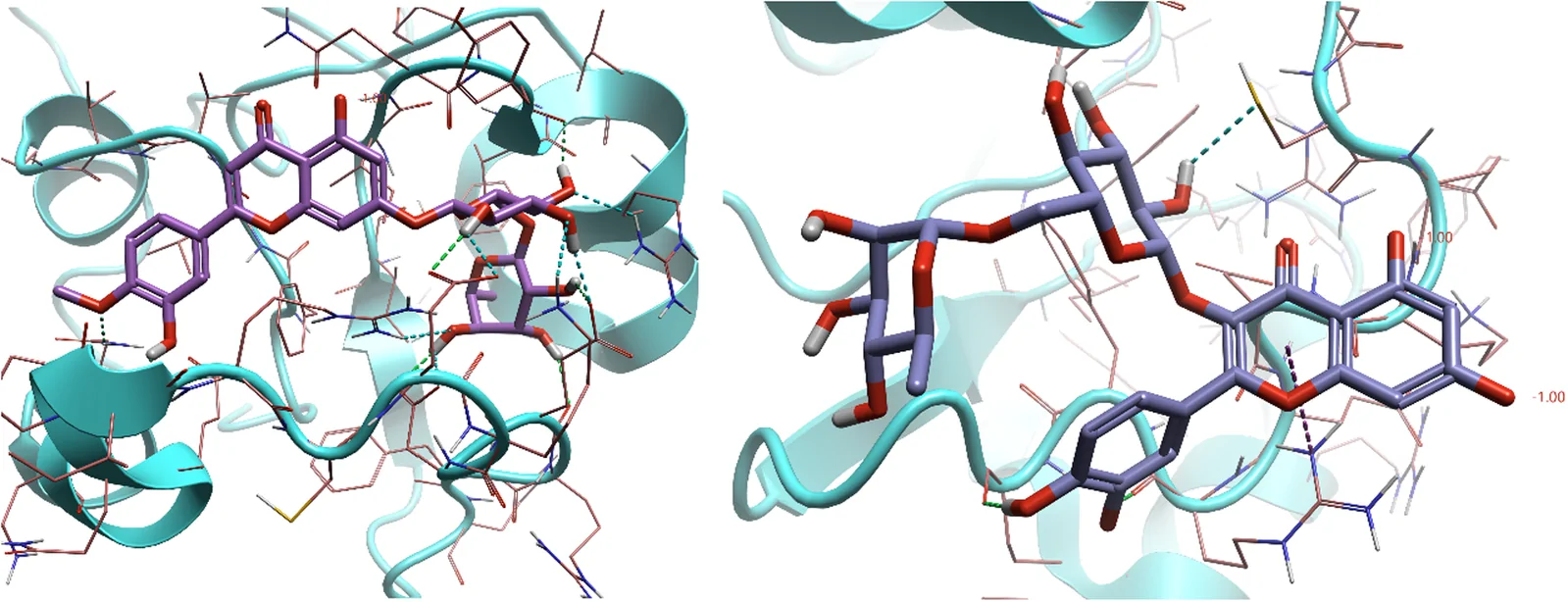
Binding topologies of diosmin (entry 2, left-hand side) and rutin (entry 4, right-hand side) to κ-casein.

**Table 11:** Binding Topological Features for Charged Diosmin and Rutin to κ-Casein Binding Pockets

**entry**	**isoform**	**binding pocket**	**molecule**	**charge**	**LF VSscore** ^a^	**interactions**
**1**	κ	k_bp1	Diosmin	0	–15.31	Q24, Q28, R31, E33, K34, F38, E36, R55 (hb)
						F6, R31, I47, Y51, V52, Y56, L61 (hc)
**2**				–1	–16.99	Q24, Q28, R31, E33, K34, D35, E36, Y51, R55 (hb)
						F6, R31, I47, Y51, V52, Y56, L61 (hc)
**3**			Rutin	–1	–10.11	Q26, C32, K34, D35, F39, S40, D41 (hb)
						R37, F39 (hc)
**4**				–2	–10.37	Q26, C32, R37, F39, D41, K42 (hb)
						R37 (cπ)

a LF VSscores are given in kcal/mol.

### 3.4 Molecular Modeling of β-Lactoglobulin–Polyphenol Interactions

To gain further insight into the experimental observations, the molecular modeling approach was extended to β-lactoglobulin, as phenolic compounds did not induce a significant reduction in IgE reactivity for β-lactoglobulin. This contrasting behavior prompted a comparative analysis aimed at evaluating whether differences in protein–ligand interactions could account for the distinct experimental responses.

Pocket scouting of the β-lactoglobulin crystal structure revealed a single prominent hydrophobic pocket with a high potential for accommodating small molecules (Figure 13). This cavity corresponds to the well-characterized central calyx of β-lactoglobulin, previously reported as the primary binding site for a variety of hydrophobic ligands, including linear fatty acids such as myristic and palmitic acids, as well as retinol. The pocket exhibited a druggability score of 0.754 (Table 12), indicating a high likelihood of supporting stable ligand binding and confirming its suitability as a principal target for docking simulations.

**Figure 13:**
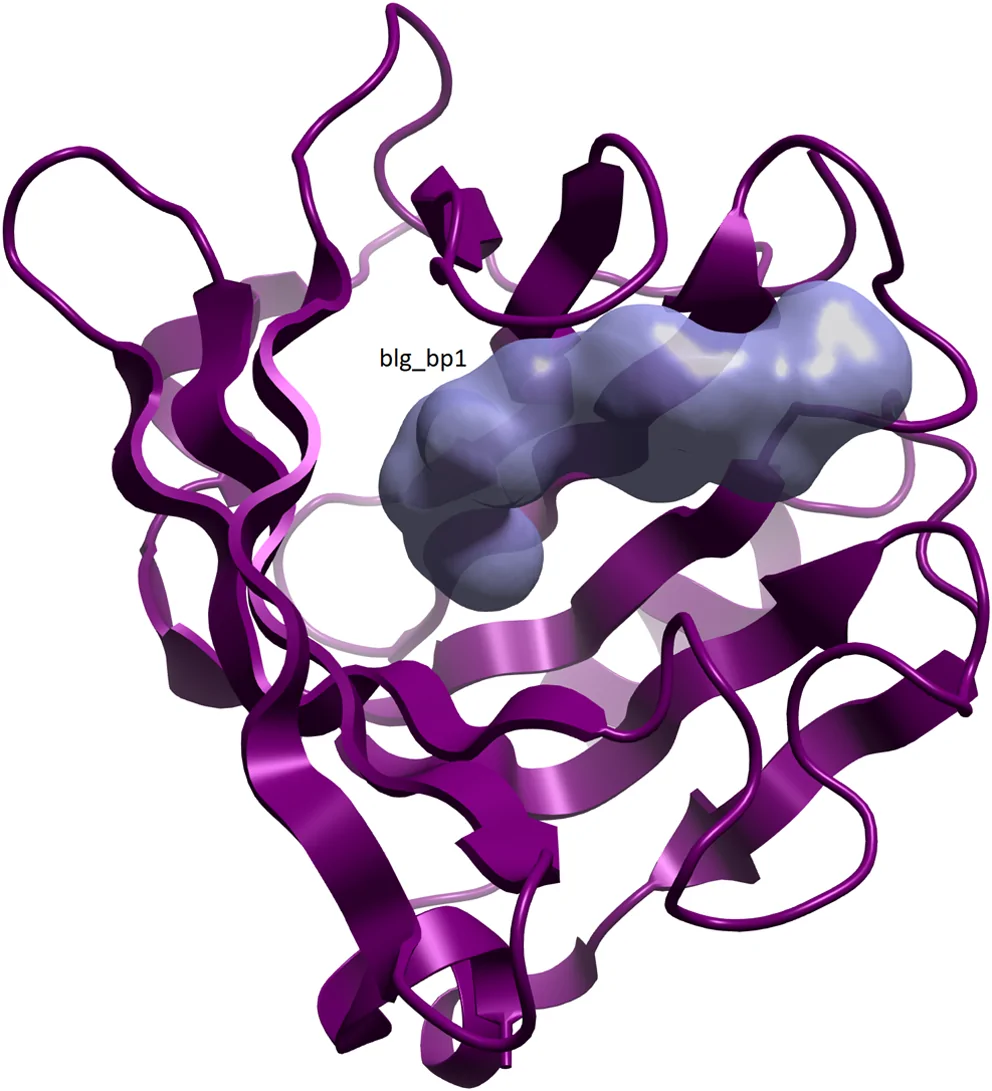
Crystal structure of β-lactoglobulin (PDB ID: 7ER3) showing the detected binding pocket blg_bp1 (the cocrystallized ligand was omitted for clarity).

**Table 12:** Binding Pocket Features of β-Lactoglobulin Binding Pocket Scouting Run

**protein**	**binding pocket**	**druggability Score**	**residues**	**epitope ID (IEDB)**
**β-lactoglobulin**	blg_bp1	0.754	PLRVYVEEL (38–46)	98700
			IDALNEKV (84–93)	
			FCMENSAEPEQSLVCQ (105–120)	74778, 22336

Diosmin and rutin also exhibit favorable binding to β-lactoglobulin, with docking scores consistent with stable ligand–protein interactions within the identified hydrophobic pocket (Figure 14). Diosmin, in its ionized form, reached an LF VSscore of −13.02 kcal/mol and adopts an extended binding pose in which the glycosidic moiety is deeply embedded within the pocket, while the bicyclic aromatic system remains partially exposed to the solvent. This orientation is stabilized by an extensive hydrogen-bonding network involving residues D28, S30, D85, N88, E108, and S116 (Table 13).

**Figure 14:**
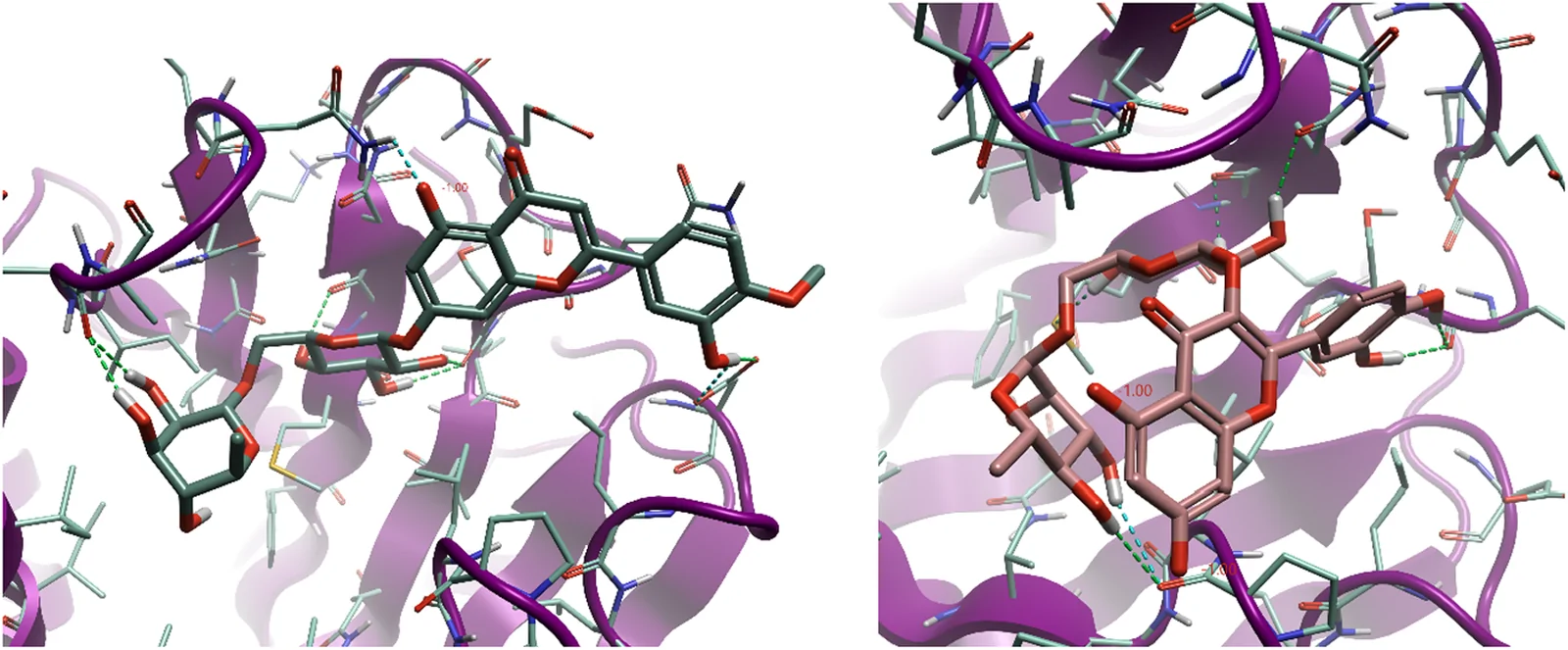
Binding topologies of diosmin (entry 2, left-hand side) and rutin (entry 4, right-hand side) to β-lactoglobulin.

**Table 13:** Binding Topological Features of Charged Diosmin and Rutin to β-Lactoglobulin Binding Pockets

**entry**	**protein**	**binding pocket**	**molecule**	**charge**	**LF VSscore** ^a^	**interactions**
**1**	β-lactoglobulin	blg_bp1	Diosmin	0	–12.87	P38, E62, K70, D85, L87, N88, N90, E112 (hb)
						I84, N109 (hc)
**2**				–1	–13.02	D28, S30, D85, N88, E108, S116 (hb)
**3**			Rutin	–1	–12.21	P38, E108, N109, Q115, S116 (hb)
						L29, P38 (hc)
**4**				–2	–12.42	P38, M107, E108, N109, Q115 (hb)
						P38, L87 (hc)

a LF VSscores are given in kcal/mol.

Rutin, in its dianionic form (−2 charge), displays a comparable binding preference, achieving its best docking score of −12.42 kcal/mol within the same pocket. The ligand adopts a more compact, folded conformation that maximizes complementarity with the binding cavity and is stabilized through hydrogen-bond interactions with P38, M107, E108, N109, and Q115, as well as hydrophobic contacts to P38 and L87 (Table 13).

In conclusion, small molecules that induce sufficient changes in folding and substrate recognition in food proteins are now a clinical reality with increasing reach over different pathologies, especially allergies. By focusing on cow’s milk proteins, in particular casein, the present study provides sound evidence that hybrid matrices composed of casein and polyphenols present in lemon peel extracts substantially reduce IgE reactivity in human sera from patients with severe allergic reactions, even life-threatening anaphylaxis, after cow’s milk consumption.

Four casein isoforms were modeled by using the I-TASSER server, reproducing previously reported structural features of bovine milk proteins. Molecular docking and binding pocket analysis were used as complementary exploratory tools to provide a structural context for experimental observations rather than as standalone predictive evidence.

The structural models are consistent with the known flexibility of caseins, showing more extended conformations for α-caseins and more compact, globular-like arrangements for β- and κ-casein. The identified binding sites span a range of hydrophobic to more polar environments, which may contribute to the observed differences in ligand interactions.

Diosmin and rutin display isoform-dependent binding tendencies, with a general preference toward more structured binding environments. For diosmin, the overall binding trend follows κ-casein > β-casein > αS1 casein ≈ αS2 casein, whereas rutin shows a preference for β-casein > κ-casein > αS1 casein ≈ αS2 casein, indicating differential affinity profiles across isoforms. Importantly, docking results for β-lactoglobulin show lower or comparable affinities relative to caseins, with diosmin exhibiting reduced binding compared to β- and κ-caseins, while rutin displays intermediate affinity between β- and κ-casein. These exploratory findings may be consistent with the absence of any reduction in IgE binding observed experimentally for β-lactoglobulin and may help to rationalize its distinct behavior compared to casein isoforms under the studied conditions. Future work should address the structural organization of casein micelles and the impact of polyphenol binding on their formation and stability, as these aspects represent the next step toward a more comprehensive understanding of casein–polyphenol interactions in the native colloidal context.

We contend that there are yet unexplored therapeutically useful mechanisms by merging food proteins with natural products that can be exploited to block/hide specific protein epitopes. It is obvious that the translation from serological assays to oral administration or other means may put lives at risk. The delivery of modified allergens in the form of a patch or snack may be easier to tolerate and require lower doses. Certainly, this area is ripe for preclinical consideration and further innovation.

## Supplementary Material



## Data Availability

All the data generated in this study are available within the article and the Supporting Information.

## Notes

Detailed methods and experimental procedures for chromatographic and spectroscopic analyses, immunological assays, and *in silico* modeling; supplemental tabulated data and copies of superimposed ATR-FTIR spectra of protein–polyphenol matrices (PDF)

## Notes

This article is dedicated *in memoriam* to Joaquín Espinosa−García and Vicente Gómez-Serrano, great friends and colleagues, top scientists, and faculty members, who left us too early.
