# Supplementary material for: Potent IgE-Mediated Allergenic Reduction in Human Serum by Casein–Polyphenol Conjugates Makes Personalized Oral Immunotherapy Credible
Source: ACS Food Sci Technol. 2026 Jul 29;6(8):2455–71. doi: 10.1021/acsfoodscitech.6c00400 (PMC13508124; doi:10.1021/acsfoodscitech.6c00400)
Supplement: Supplementary file 1 [file fs6c00400_si_001.pdf]

Supporting Information

Potent IgE-Mediated Allergenic Reduction in Human Serum by Casein-Polyphenol Conjugates Makes Personalized Oral Immunotherapy Credible

M. Victoria Gil,* Nuria Fernández-Rivera, Gloria Gutiérrez-Díaz, Jorge Parrón-Ballesteros, Carlos Pastor-Vargas, Diana Betancor, Carlos Nieto, and Pedro Cintas

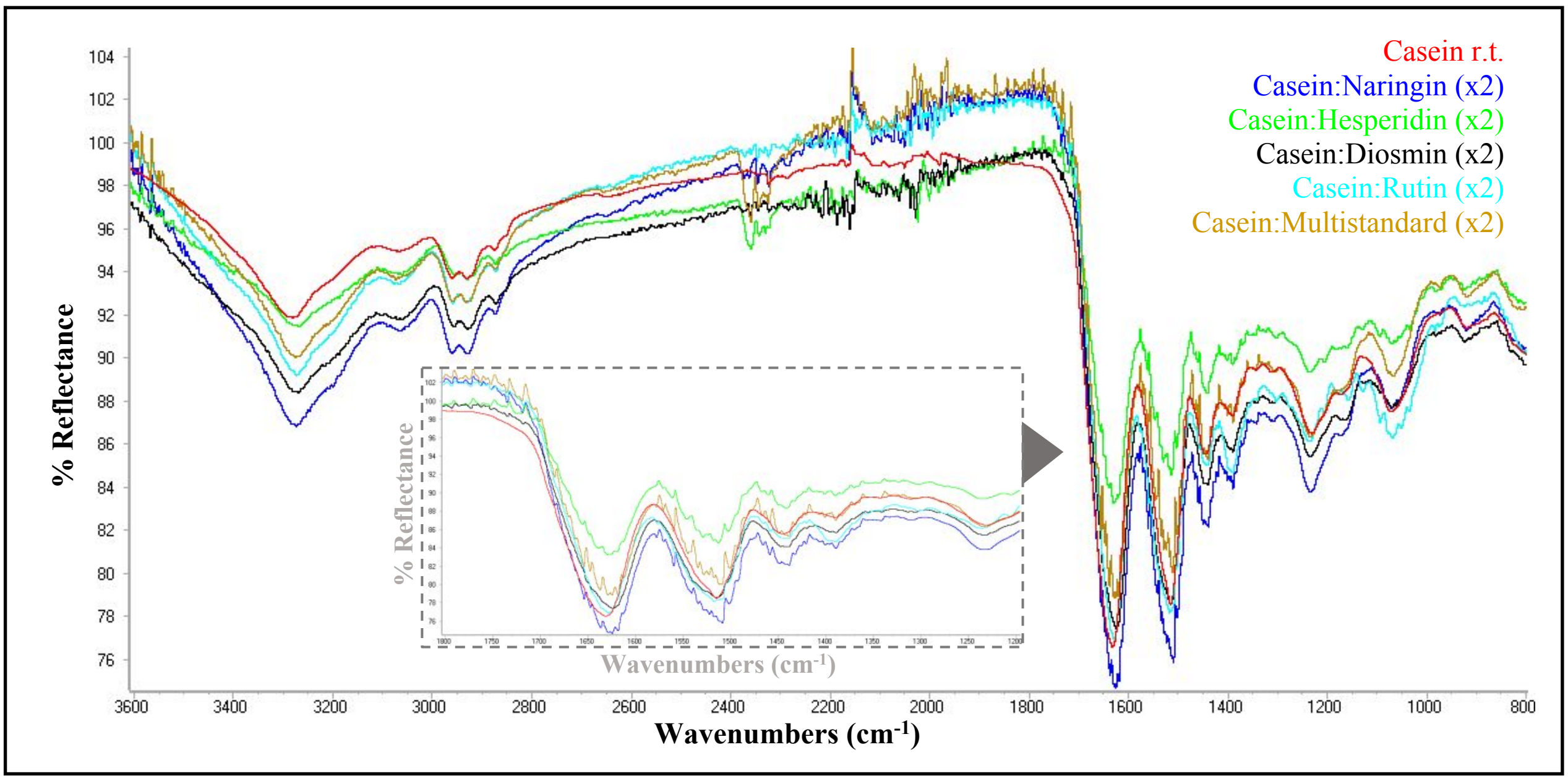

Figure S1. FTIR spectra (ATR mode) of casein and its matrices with pure polyphenols and a multistandard solution with double concentrations of phenolics. The inner inset shows an enlargement of the peaks corresponding to amide bands I and II (1800-1200 cm^{-1}).

Table S1. Quantitative Analysis of Phenolics in Supernatants After Matrix Formation[a]

Polyphenol		Concentration in starting solution (mg/L)[b]	Concentration in supernatant (mg/L)[b]	Interaction (%)
**Naringin**		5.159 ± 0.023	5.089 ± 0.027*	1.4
**Hesperidin**		87.910 ± 0.130	38.922 ± 0.112*	55.7
**Diosmin**		0.920 ± 0.010	0.065 ± 0.006*	85.5
**Rutin**		1.129 ± 0.028	0.308 ± 0.013*	72.7
**Multistandard**	Naringin	5.143 ± 0.018	5.907 ± 0.022*	-
	Hesperidin	87.831 ± 0.028	38.692 ± 0.095*	55.9
	Diosmin	0.918 ± 0.012	0.160 ± 0.026*	82.6
	Rutin	1.135 ± 0.009	0.141 ± 0.002*	87.6

[a]Determined by HPLC analyses in solutions containing double concentration of polyphenols with respect to that measured in lemon peel extracts. [b]Data expressed as mean ± standard deviation. [c]Significant differences ($p < 0.05$) relative to the initial concentrations.

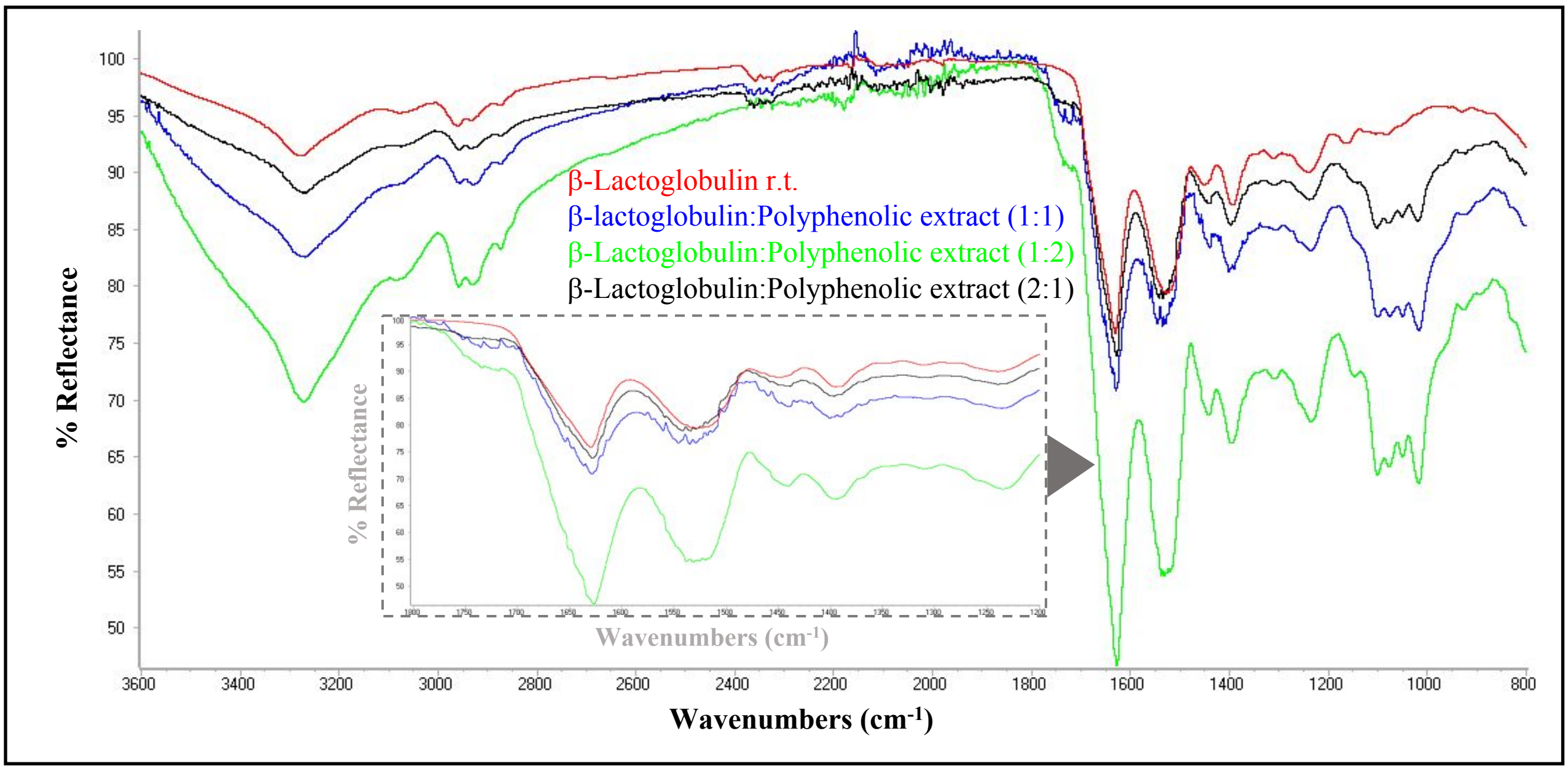

Figure S2. Superimposed FTIR spectra (ATR mode) of unmodified β-lactoglobulin and its matrices with polyphenols from lemon peels at room temperature. The inner inset shows an enlargement of the absorptions corresponding to amide bands I and II (1800-1200 cm^{-1}).

Table S2. Identification and Quantitative Analysis of Polyphenols from Lemon Peel Extract (Supernatant Phase) After Matrix Formation with β-Lactoglobulin[a]

Polyphenol	Concentration in extract (mg/g extract)[b]	Concentration in supernatant (mg/g extract)[b]	Interaction (%)
		**Ratio 1:1**	
**Naringin**	0.563 ± 0.038	0.547 ± 0.002	2,9*
**Hesperidin**	9.662 ± 0.087	4.044 ± 0.016*	58.1
**Eriocitrin**	2.627 ± 0.071	1.130 ± 0.019*	57.0
**Rutin**	0.130 ± 0.023	0.084 ± 0.009*	35.4
**Diosmin**	0.115 ± 0.011	0.056 ± 0.006*	51.3
		**Ratio 1:2**	
**Naringin**	0.563 ± 0.038	0,558 ± 0.003	0.8
**Hesperidin**	9.662 ± 0.087	4.350 ± 0.051*	51.9
**Eriocitrin**	2.627 ± 0.071	1.291 ± 0.071*	50.9
**Rutin**	0.130 ± 0.023	0.094 ± 0.009	27.7
**Diosmin**	0.115 ± 0.011	0.061 ± 0.003*	34.8
		**Ratio 2:1**	
**Naringin**	0.563 ± 0.038	0.454 ± 0.017*	19.4
**Hesperidin**	9.662 ± 0.087	2.122 ± 0.153*	78.0
**Eriocitrin**	2.627 ± 0.071	0.554 ± 0.048*	78.9
**Rutin**	0.130 ± 0.023	0.042 ± 0.006*	67.7
**Diosmin**	0.115 ± 0.011	0.024 ± 0.004*	79.1

[a]Determined by HPLC analyses. [b]Data expressed as mean ± standard deviation. [c]Significant differences ($p < 0.05$) relative to the initial concentrations.

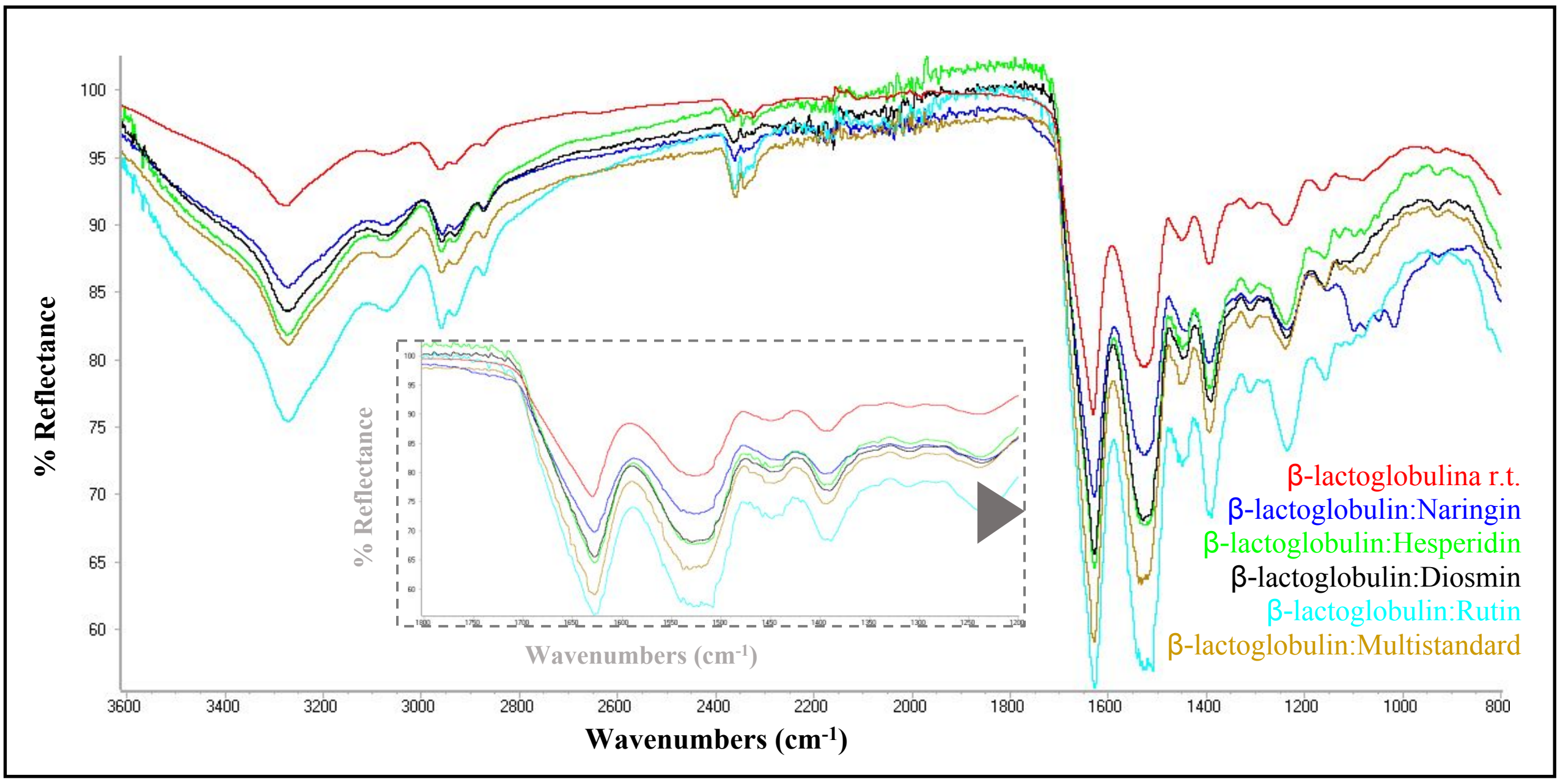

Figure S3. FTIR spectra (recorded in ATR mode) of β-lactoglobulin and its matrices with pure polyphenols and a multistandard solution. The inset depicts an enlargement of the bands corresponding to amide bands I and II (1800-1200 cm^{-1}).

Table S3. Quantitative Chromatographic Analysis of Polyphenols in Supernatants After Matrix Formation[a]

Polyphenol		Concentration in starting solution (mg/L)[b]	Concentration in supernatant (mg/L)[b]	% Interaction
**Naringin**		2.502 ± 0.067	2.493 ± 0.005	0.4
**Hesperidin**		43.852 ± 0.043	28.061 ± 0.085*	47.4
**Diosmin**		0.463 ± 0.006	0.061 ± 0.038*	86.8
**Rutin**		0.548 ± 0.017	0.063 ± 0.018*	88.5
**Multistandard**	Naringin	2.599 ± 0.081	3.090 ± 0.029*	-
	Hesperidin	43.825 ± 0.034	23.921 ± 0.058*	45.4
	Diosmin	0.474 ± 0.012	0.136 ± 0.006*	71.3
	Rutin	0.570 ± 0.017	0.058 ± 0.042*	89.8

[a]Determined by HPLC analyses. [b]Data expressed as mean ± standard deviation. [c]Significant differences ($p < 0.05$) relative to the initial concentrations.

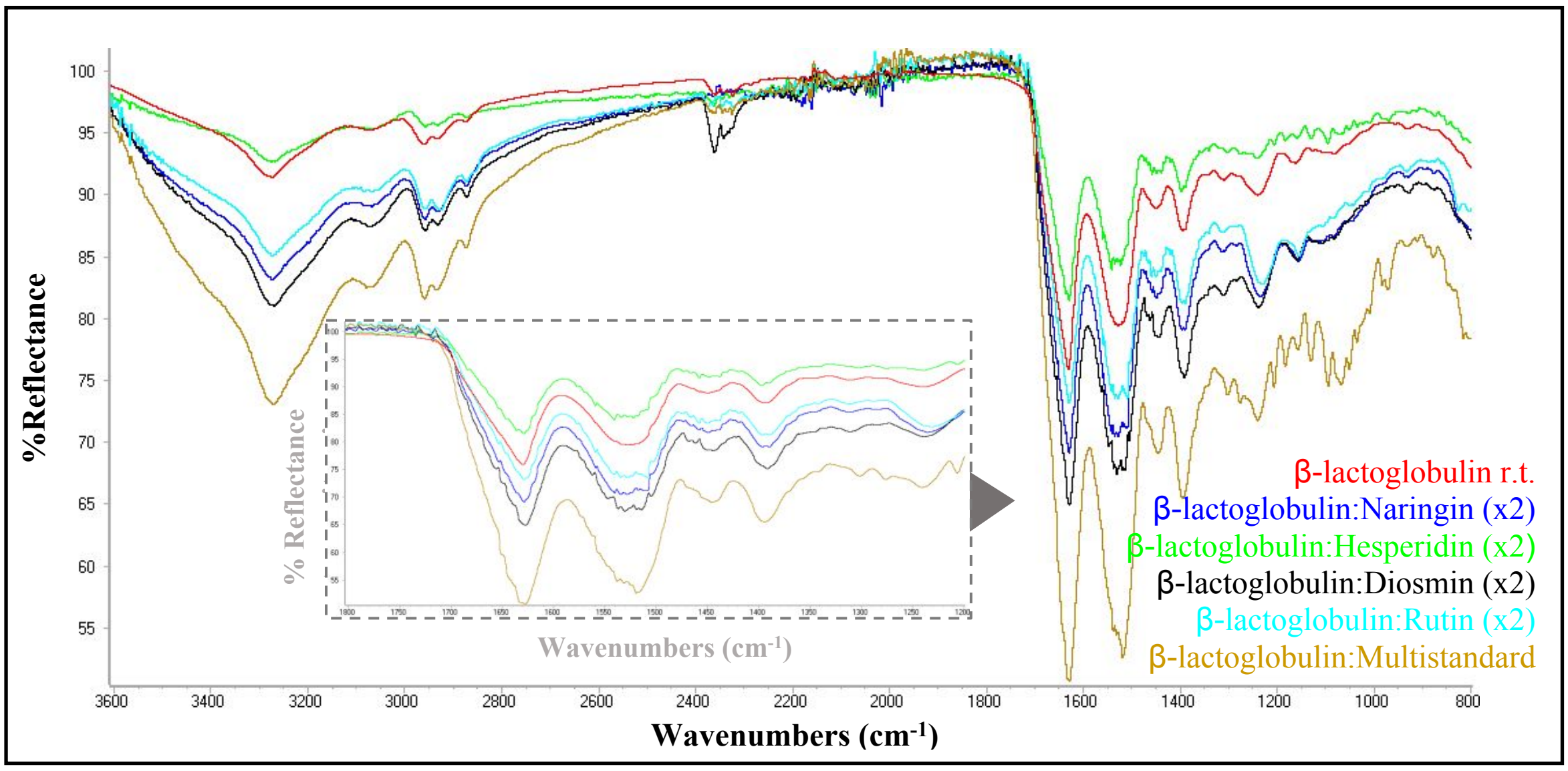

Figure S4. Superimposed FTIR-ATR spectra of β-lactoglobulin and its matrices with pure polyphenols and a multistandard solution with double concentrations of phenolics. The inset shows a magnification of the broad zone comprising amide bands (1200-1800 cm^{-1}).

Table S4. Quantitative HPLC Analysis of Phenolics in Supernatants After Matrix Formation[a]

Polyphenol		Concentration in starting solution (mg/L)[b]	Concentration in supernatant (mg/L)[b]	% Interaction
**Naringin**		5.159 ± 0.023	5.152 ± 0.004	0.1
**Hesperidin**		87.910 ± 0.130	27.120 ± 0.180*	69.1
**Diosmin**		0.920 ± 0.010	0.207 ± 0.012*	77.5
**Rutin**		1.129 ± 0.028	0.116 ± 0.008*	89.7
**Multistandard**	Naringin	5.143 ± 0.018	6.044 ± 0.084*	-
	Hesperidin	87.831 ± 0.028	28.765 ± 0.033*	67.3
	Diosmin	0.918 ± 0.012	0.131 ± 0.008*	85.7
	Rutin	1.135 ± 0.009	0.136 ± 0.007*	88.0

[a]Determined by HPLC analyses. [b]Data expressed as mean ± standard deviation. [c]Significant differences ($p < 0.05$) relative to the initial concentrations.
